# Adipose Tissue Modification through Feeding Strategies and Their Implication on Adipogenesis and Adipose Tissue Metabolism in Ruminants

**DOI:** 10.3390/ijms21093183

**Published:** 2020-04-30

**Authors:** Olaia Urrutia, José Antonio Mendizabal, Leopoldo Alfonso, Beatriz Soret, Kizkitza Insausti, Ana Arana

**Affiliations:** IS-FOOD Institute, Escuela Técnica Superior de Ingeniería Agronómica y Biociencias, Departamento de Agronomía, Biotecnología y Alimentación, Universidad Pública de Navarra, 31006 Pamplona, Spain; jamendi@unavarra.es (J.A.M.); leo.alfonso@unavarra.es (L.A.); soret@unavarra.es (B.S.); kizkitza.insausti@unavarra.es (K.I.); aarana@unavarra.es (A.A.)

**Keywords:** *n*-3 polyunsaturated fatty acids (PUFA), adipose tissue, lipid metabolism, adipocytes, adipogenesis, lipogenesis

## Abstract

Dietary recommendations by health authorities have been advising of the importance of diminishing saturated fatty acids (SFA) consumption and replacing them by polyunsaturated fatty acids (PUFA), particularly omega-3. Therefore, there have been efforts to enhance food fatty acid profiles, helping them to meet human nutritional recommendations. Ruminant meat is the major dietary conjugated linoleic acid (CLA) source, but it also contains SFA at relatively high proportions, deriving from ruminal biohydrogenation of PUFA. Additionally, lipid metabolism in ruminants may differ from other species. Recent research has aimed to modify the fatty acid profile of meat, and other animal products. This review summarizes dietary strategies based on the *n*-3 PUFA supplementation of ruminant diets and their effects on meat fatty acid composition. Additionally, the role of *n*-3 PUFA in adipose tissue (AT) development and in the expression of key genes involved in adipogenesis and lipid metabolism is discussed. It has been demonstrated that linseed supplementation leads to an increase in α-linolenic acid (ALA) and eicosapentaenoic acid (EPA), but not in docosahexaenoic acid (DHA), whilst fish oil and algae increase DHA content. Dietary PUFA can alter AT adiposity and modulate lipid metabolism genes expression, although further research is required to clarify the underlying mechanism.

## 1. Introduction

Dietary recommendations by Food and Agriculture Organization of the United Nations/World Health Organization (FAO/WHO), as well as other European health authorities, advise replacing saturated fatty acids (SFA) with polyunsaturated fatty acids (PUFA) and increasing the intake of omega 3 or *n*-3 PUFA, thereby decreasing the *n*-6/*n*-3 ratio. Thus, *n*-3 PUFA have received great interest, especially long-chain PUFA (LCPUFA), such as docosahexaenoic acid (DHA) (C22:6*n*-3) and eicosapentaenoic acid (EPA) (C20:5*n*-3), due to its potential benefits for human health [1,2]. Fatty acids are important constituents of cellular membranes and they have important biological and structural roles, in addition to their role as a source of energy [3]. DHA is a key component of cellular membranes, and is essential for maintaining the structure and function of the brain [4]. Furthermore, DHA and EPA are important for the generation of biologically active DHA- and EPA- derived lipid mediators in white adipose tissue (AT), which exert mostly anti-inflammatory effects [5].

Adipose tissue is the principal site of de novo fatty acid synthesis in ruminants and adipogenesis and lipid metabolism in these meat animals may/may not be similar to other animal species [6]. Meat is a significant source of protein, which is of high biological value. In addition, it contains micronutrients, such as minerals (iron, selenium or zinc, among others), vitamins (B6, B12, A, D) and bioactive lipids [7,8]. Ruminant meat presents a relatively high proportion of SFA and variable content of *trans* fatty acids, which are the result of ruminal biohydrogenation of PUFA from the diet [7,8]. However, meat from ruminant animals is the main dietary conjugated linoleic acid (CLA) source, including the C18:2*c*9*t*11 isomer, which is known for its potential health benefits [9].

Over the past decade, consumer’s demands regarding food products have changed towards products that are safe, nutritious, and of good eating quality, but that are also affordable and produced through sustainable methods, which shows the complexity of the current consumer behavior [10]. This applies as well to meat and, therefore, meat production is dealing with challenges such as environmental sustainability (for instance, the need to reduce greenhouses gases emissions), societal (for instance, the need for animal welfare) and health issues (for instance, the need to reduce SFA consumption). Some international organizations and recent studies conclude that the consumption of meat, particularly red meat, should be reduced, in order to achieve a healthy and sustainable diet. Nevertheless, this also implies the promotion and maintenance of sustainable animal production systems, which provide different ecosystem services, in addition to nutritious food and high biological value proteins. In this contest, one of the most important factors influencing the changes in consumer’s demands for meat and meat products are health concerns [11], so research aiming to modify meat fatty acid profiles through different animal feeding strategies has been intensified, in order to obtain meat in compliance with the current human nutritional recommendations [12,13,14,15]. There are a wide variety of feeds that are commonly used to modify the composition of PUFA in meat obtained from ruminants. These ingredients can be forages of different types, vegetable oils (such as sunflower, soja, canola, etc.), oilseeds (such as linseed and canola), and fish oils and marine algae [8]. Linseed is one of the most studied seeds due to its high α-linolenic acid (ALA) content (C18:3*n*-3) (56%), and it is used to promote the biosynthesis of *n*-3 LCPUFA, such as EPA and DHA, and to enrich meat in *n*-3 PUFA, as ALA is the precursor for the endogenous synthesis of these fatty acids. Furthermore, marine microalgae or fish oil are the main natural sources of EPA and DHA, and they are used as another feeding strategy.

To perform such modification, it is important to deepen the knowledge of the development and metabolism of the AT, as well as the changes induced by PUFA supplementation in ruminant diets. Research with meat purpose animals may help to obtain a better understanding of the regulation of lipid metabolism and adipocyte physiology, which may serve to improve meat quality and also human health, particularly in individuals with lipodystrophy and obesity.

This review focuses on highlighting some dietary strategies based on the inclusion of *n*-3 PUFA in the diet of ruminants. Their effects on the fatty acid composition of meat, on the AT development and on the expression of key genes involved in adipogenesis and lipid metabolism are reviewed. A brief description of PUFA metabolism in the rumen, as well as lipid metabolism in ruminants, is also presented.

## 2. Metabolism of PUFA in Ruminants

### 2.1. PUFA Metabolism in the Rumen

The dietary lipids are transformed in the rumen by microbes via two processes: biohydrogenation and lipolysis. Ruminal biohydrogenation is the microbial saturation of dietary unsaturated fatty acids, which limits the availability of health-related PUFA in ruminant meat and milk. Consequently, in spite of significant amounts of PUFA entering the rumen, outflows of PUFA into the small intestine are limited.

The lipolysis process is considered an important process, because it is a prerequisite for the subsequent PUFA biohydrogenation in the rumen. In this process, lipases, phospholipases and microbial galactolipases hydrolyze the ester bonds of complex lipids (triacylglycerides (TG), phospholipids (PL) and galactolipids), leading mainly to non-esterified fatty acids and glycerol, in addition to the amino acids (derived from PL) and galactose (derived from galactolipids). In most cases, the hydrolysis of dietary lipids is very high, in the range of 85–95% [16], however, this process can be affected by several factors. Lipolysis can be reduced by the addition of antibiotics [17] or by a decrease in ruminal pH (pH ≤ 6.0) [18], as has been observed in in vitro studies. Other factors that cause lowering lipolysis are the increase in fat level in the diet, and the number of double bonds of fatty acids.

After lipolysis, part of the resulting PUFA (70–90%) undergo the microbial biohydrogenation, and the dietary PUFA are transformed to SFA. First, an isomerization occurs, by which *trans* intermediates are formed, followed by hydrogenation of the double bonds. As a consequence, the metabolism of PUFA in the rumen results in the synthesis of stearic acid (C18:0), this being the main fatty acid that reaches the duodenum [19].

**Figure 1:**
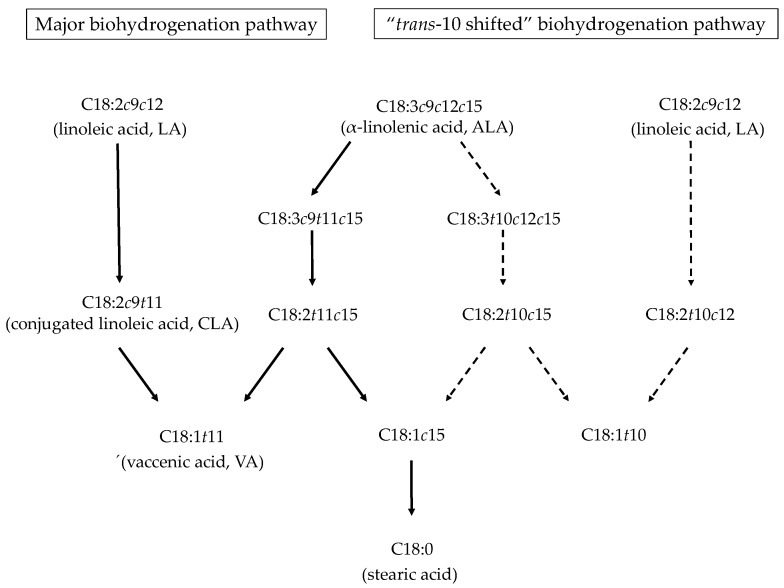
Description of linoleic acid (LA) and α-linolenic acid (ALA) biohydrogenation pathways in the rumen. The arrows with a continuous line describe the major pathway, and the dashed lines describe the plausible pathway proposed by Griinari and Bauman [20]. Adapted from Alves and Bessa [21].

The sequence of the biohydrogenation of linoleic acid (LA) and ALA is shown in Figure 1. During the biohydrogenation of LA, the first step is the isomeration of the *cis*-12 double bond to *trans*-11, which results in the synthesis of some CLA isomers (C18:2*c*9*t*11; C18:2*t*9*c*11; C18:2*t*10*c*12; etc.). The predominant fatty acid is the isomer C18:2*c*9*t*11, called rumenic acid. Subsequently, the *cis*-9 double bond is hydrogenated, resulting in the conversion of CLA to C18:1*t*11 (vaccenic acid, VA), followed by hydrogenation to stearic acid (C18:0). Similar to LA, the biohydrogenation of ALA begins with an isomeration of the *cis*-12 bond, with C18:3*c*9*t*11*c*15 being the most abundant product, although other intermediate products, such as C18:3*t*9*t*11*c*15 or C18:3*c*9*t*13*c*15, are also formed. In a second step, the *cis*-9 and *cis*-15 bonds are hydrogenated by two consecutive steps resulting in VA, and finally, hydrogenated to stearic acid [19].

Biohydrogenation of PUFA in the rumen occurs at a high extent. For instance, more than 85% of dietary ALA is hydrogenated in the rumen. Similarly, more than 90% of dietary EPA and DHA from fish oil are not recovered in the duodenum, although algae products might be much less hydrogenated [13].

Griinari and Bauman [20] proposed the “biohydrogenation theory”, which states that when the amount of highly fermentable grains (i.e., concentrate) is added to the diet is high, a change in the bacterial population occurs, and ruminal fermentation patterns are altered. Consequently, LA and ALA are metabolized through an altered step (*trans*-10 shifted). LA is transformed to C18:2*t*10*c*12 by isomerization and subsequently reduced to C18:1*t*10, while ALA appears to be transformed to C18:3*t*10*c*12*c*15 and C18:3*t*10*c*15, and finally to C18:1*t*10.

### 2.2. Lipogenesis

**Figure 2:**
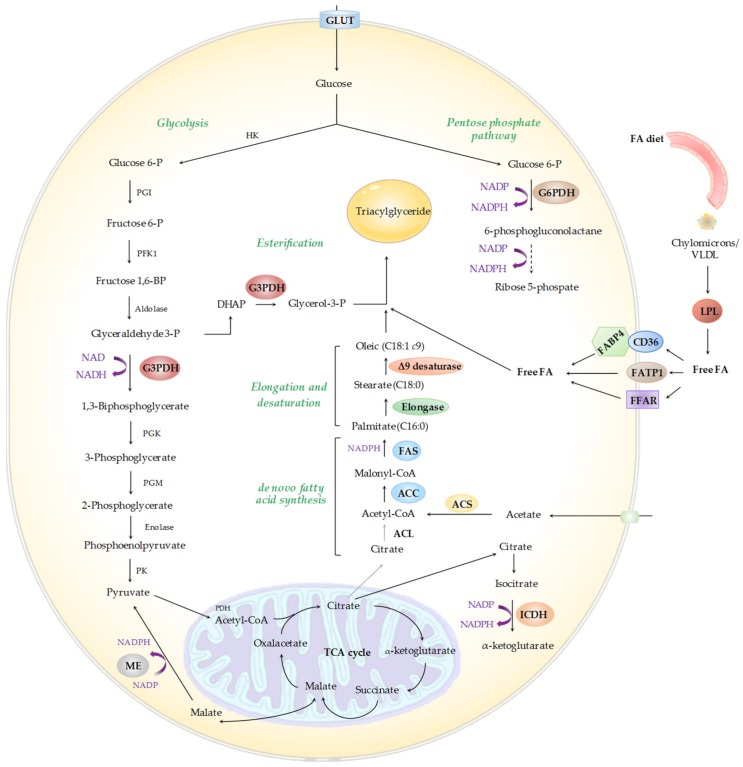
Schematic representation of the metabolic pathways involved in the synthesis of fatty acids and triacylglycerides. Adapted from Vander Heiben et al. [26] and Ladeira et al. [25]. ACC = acetyl-CoA carboxylase; ACL = ATP-citrate lyase; ACS = acyl-CoA synthetase; CD36 = fatty acid translocase; FA = fatty acid; FABP4 = fatty acid-binding protein 4; FAS = fatty acid synthase; FATP1 = fatty acid transport protein 1; FFAR = free fatty acid receptor; G3PDH = glycerol-3-phosphate dehydrogenase; G6PDH = glucose-6-phosphate dehydrogenase; GLUT = glucose transporter; HK = hesoxe kinase; ICDH = isocitrate dehydrogenase; LPL = lipoprotein lipase; ME = malic enzyme; PDH = pyruvate dehydrogenase; PFK1 = phosphofructose kinase; PGI = phosphoglucose isomerase; PGK = phosphoglycerate kinase; PGM = phosphoglycerate mutase; PK = pyruvatekinase; TCA = tricarboxylic acid cycle; VLDL = very low density lipoproteins.

After ruminal biohydrogenation, the lipids available for absorption in the small intestine include mainly SFA (stearic acid and palmitic acid, C16:0), biohydrogenation intermediates and microbial PL, in addition to dietary by-pass TG [12,22]. Absorbed lipids are re-esterified to newly formed TG and PL. Subsequently, TG, PL, cholesterol and apoproteins are used to synthesize chylomicrons, which, along with very low density lipoproteins (VLDL), are secreted to the lymph, and then to the general blood stream through the thoracic duct [23]. In ruminants, intestinally synthesized VLDL predominates, whilst chylomicrons would be more important in the milk-fed preruminants [24]. In the peripheral tissues (principally skeletal muscle, AT and mammary gland), lipoprotein lipase (LPL) hydrolyzes TG found in chylomicrons and VLDL (Figure 2). Afterwards, fatty acids are transported into the cells by four groups of fatty acid transporters: fatty acid translocase (CD36), fatty acid transport protein (FATP), fatty acid-binding protein (FABP) in association with acyl-CoA synthethase or free fatty acid receptors (FFARs) [25]. After uptake, fatty acids are bound to fatty acid binding proteins, such as FAPB4, and can undergo different metabolic fates, including oxidation in mitochondria or esterification and storage in lipid droplets.

As said above, fatty acids derived from the diet and those synthesized de novo in adipocytes are stored in the form of TG in lipid droplets. As shown in Figure 2, TG are synthesized by esterification of fatty acid acyl chains and glycerol-3-phosphate (G3P), which derives from dihydroxyacetone phosphate (DHAP) through the glycerol-3-phosphate dehydrogenase (G3PDH) enzyme.

Fatty acid biosynthesis de novo is undertaken by successive enzymatic reactions, which include key enzymes, such as ATP-citrate lyase (ACL), acyl-CoA synthethase (ACS), acetyl-CoA carboxylase (ACC), the fatty acid synthase (FAS) complex, fatty acid elongase (ELOVL) and Δ9 desaturase or stearoyl-CoA desaturase (*SCD*) [27]. In ruminants, acetate (in less proportion propionate and butyrate), but not glucose, is the principal precursor of de novo lipogenesis within the tissues, including AT, and usually the low activity of ACL and malate dehydrogenase accounted for this phenomenon [25]. The necessary hydrogen sources for the synthesis of fatty acids can be formed through different metabolic pathways. Nicotinamide adenine dinucleotide phosphate (NADPH) formed during the process of glucose oxidation through the pentose phosphate pathway is used as hydrogen donor. The enzymes responsible for NADPH formation are glucose-6-phosphate dehydrogenase (G6PDH) and 6-phosphogluconate dehydrogenase (6PGD). The NADPH is also formed by the enzyme isocitrate dehydrogenase (ICDH), responsible for the conversion of isocitrate to α-ketoglutarate. Finally, when malate is oxidatively decarboxylated by NADP-malate dehydrogenase or malic enzyme (ME) to form pyruvate, CO_2_ and NADPH are formed [28].

Fatty acids synthesized de novo and those obtained from the diet are modified through desaturation and elongation on the endoplasmatic reticulum membrane. Palmitoleic acid (C16:1*c*9) and oleic acid (C18:1*c*9) are the major fatty acids in cells, which are synthesized from palmitic acid and stearic acid, respectively. A key enzyme involved in this process is the Δ9 desaturase enzyme (encoded by *SCD* gene). This enzyme introduces a double *cis* bond between carbons 9 and 10, and it is the rate-limiting enzyme in the synthesis of monounsaturated fatty acids (MUFA) from SFA. Furthermore, Δ9 desaturase is also responsible for the synthesis of CLA (C18:1*c*9*t*11) from VA (C18:1*t*11) in the AT, the latter formed in ruminal biohydrogenation [29,30]. The fatty acids shynthesized by Δ9 desaturase are the major MUFA of membrane PL, TG, and cholesterol esters. Therefore, the expression and activity of the enzyme is of great importance in the fatty acid composition of AT, regulating membrane fluidity and lipid metabolism, as previously described in mouse models [31].

**Figure 3:**
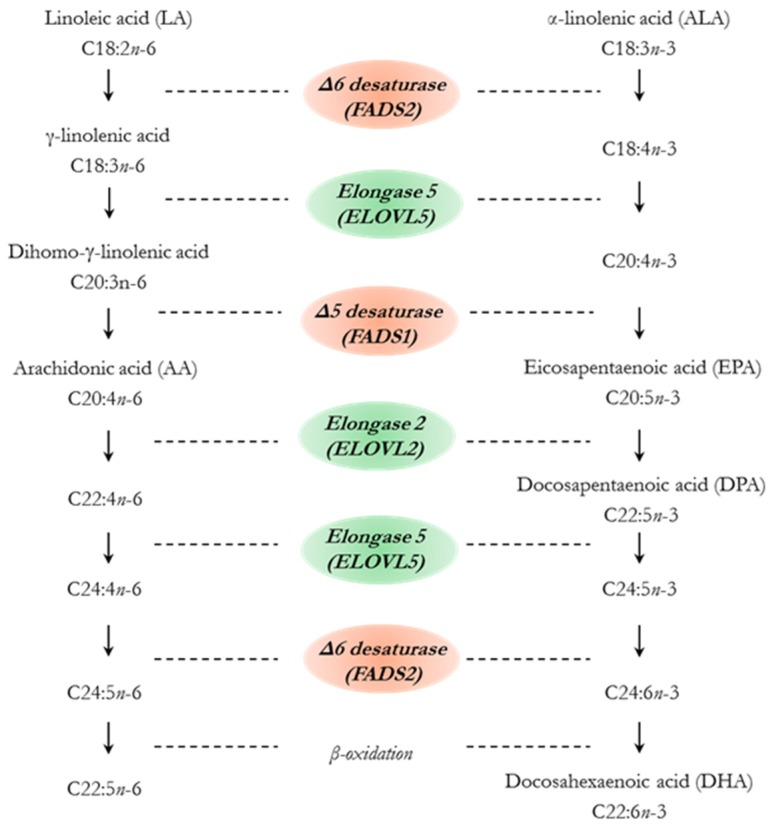
*n*-6 and *n*-3 long chain polyunsaturated fatty acid (LCPUFA) biosynthesis pathways from linoleic acid (LA) and α-linolenic acid (ALA) in animals, including ruminants. Adapted from Pereira et al. [33]. *ELOVL2* and *5* = *fatty acid elongase 2* and *5; FADS1* and *2* = *fatty acid desaturase 1* and *2.*

The essential fatty acids LA and ALA are also converted to their longer chain homologues by a combination of subsequent reactions in the microsomal fraction of the endoplasmic reticulum (Figure 3). Nevertheless, in ruminants as in other mammals, this process is inefficient and most LCPUFA in tissues derive from the diet [4,32].

The fatty acid LA is metabolized to arachidonic acid (AA) (20:4*n*-6) through a series of desaturation and elongation reactions. Firstly, LA is desaturated by Δ6 desaturase (fatty acid desaturase 2 gene, *FADS2*) to γ-linolenic acid (18:3*n*-6), which is then elongated by elongase 5 (*ELOVL5* gene) to di-homo-γ-linolenic acid (C20:3*n*-6) and subsequently desaturated by Δ5 desaturase (*FADS1* gene) to AA. Through additional elongations by elongase 2 (*ELOVL2* gene) and elongase 5 (*ELOVL5* gene), a desaturation by Δ6 desaturase (*FADS2* gene) and a final step of peroxisomal β-oxidation AA is converted to C22:5*n*-6.

On the other hand, ALA can be converted to EPA (20:5*n*-3) and DHA (22:6*n*-3) through the same series of reactions. ALA is desaturated by Δ6 desaturase to 18:4*n*-3, then subsequently elongated by elongase 5 to 20:4*n*-3, and finally desaturated by Δ5 desaturase to EPA. Through additional two elongations (elongase 2 and elongase *5*), a desaturation (Δ6 desaturase) and a final step of peroxisomal β-oxidation EPA can be converted to DHA [32]. It is of note that the rate limiting step in this metabolic pathway is Δ6 desaturase (*FADS2* gene). Both fatty acids of the *n*-6 and *n*-3 families are metabolized by this enzyme, although the desaturase has higher affinity for *n*-3 than *n*-6 PUFA (2–3 times higher for ALA than for LA), as it has described in humans, rats or mice [34,35]. However, the presence of large amounts of LA in the diet can shift the enzymatic preference of *n*-3 family fatty acids [36].

### 2.3. Adipogenesis

**Figure 4:**
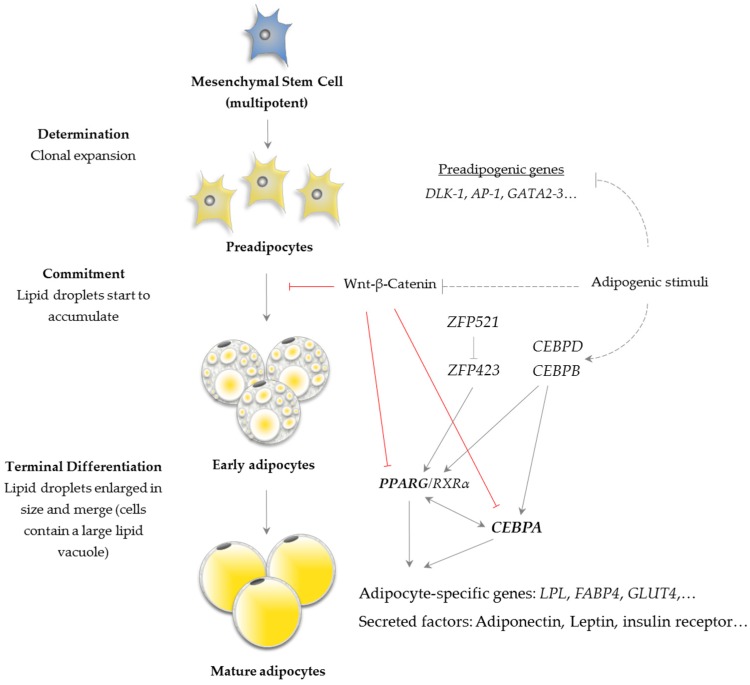
Schematic representation of the transition process from mesenchymal stem cell to mature adipocytes and transcriptional cascade during adipogenesis [38]. *DLK-1* = *delta-like 1 homolog*; *AP-1 = activator protein-1*; *GATA2* and *3* = *GATA-binding proteins 2* and *3*; *WNT* = *wingless-type MMTV integration site family members*; *ZFP423* = *zinc finger protein 423*; *CEBPA*, *B* and *D* = *CCAAT/enhancer-binding protein alpha, beta* and *delta; PPARG* = *peroxisome proliferator-activated receptor gamma*; *RXRA* = *retinoic X receptor alpha*; *LPL* = *lipoprotein lipase*; *FABP4* = *fatty acid-binding protein 4* and *GLUT4* = *glucose transporter type 4*.

Adipogenesis is the process by which multipotent mesenchymal precursor cells differenciate into mature lipid-filled adipocytes [37]. In vitro studies have described that the differentiation of preadipocytes to adipocytes occurs in two stages (determination and terminal differentiation) and implicates a comprehensive network including transcription factors responsible for expression of key proteins that induce mature adipocyte formation (Figure 4).

In the first stage of determination, the pluripotent stem cell takes on the characteristics of the adipocyte lineage and involves transformation of the stem cell into a preadipocyte that morphologically is similar to its precursor. During this transition, the cell loses its ability to turn into other types of cells [39]. Both positive (*zinc finger protein 423* or *ZFP423*; *activator protein-1* or *AP-1*) and negative (*delta-like 1 homologue* or *DLK1*, *GATA-binding proteins 2* and *3* and *wingless-type MMTV integration site family members* or WNTs) regulatory factors are known to take part in the initial stage. Adipogenesis is a complex process regulated by a wide number of transcription factors and regulators, therefore, there is still ongoing research in this area, and additional transcriptional factors and genes involved in this process continue to be identified [40].

The terminal differentiation involves changes in cell morphology, induction of insulin sensibility and changes in secretory capacity [37]. This is accompanied by the accumulation of fat inside the cell and a change in morphology into a more globular shape [41]. This second stage is characterized by a cascade of transcriptional events in which the first wave consists of induction of *CCAAT/enhancer-binding protein* (*CEBP*) *beta* (*CEBPB*) and *delta* (*CEBPD*), which activate the expression of the central adipogenesis factors *peroxisome proliferator-activated receptor gamma* (*PPARG*) and *CEBP alpha (CEBPA)*. As described in NIH-3T3 mouse cells lines, these transcription factors stimulate expression of genes involved in lipid metabolism, such as *LPL*, *FABP4* and *glucose transporter type 4* (*GLUT4*) [42,43].

A variety of extracellular factors can regulate of the above-mentioned transcription factors and they are able to determine whether preadipocytes start the process of differentiation or remain quiescent. The activating factors include insulin, glucocorticoids, prostaglandins and also MUFA and PUFA, which appear to be *PPARG* activators [44].

Fat deposited within the muscles known as intramuscular (IM) fat or marbling, along with muscle fibers and connective tissue, play key roles in the determination of meat quality. Not only in terms of nutrition related to the type of fatty acids, but also linked to tenderness, juiciness and flavor of the meat [38]. In addition to intramuscularly, fat is present in meat in the form of intermuscular fat, subcutaneous (SC) fat and membrane PL.

Generally, fat deposition increases with animal weight and age, but the development of the different fat depots is not uniform from either a quantitative or a temporal standpoint. Greater rates of fat accretion in cattle first occurs around the kidney’s knob and channel fat, followed by deposition in intermuscular and SC depots and, finally, IM depot [38]. Nevertheless, in sheep, intermuscular fat matures earlier than SC fat and internal fat depots [45]. On the other hand, goats store fat around internal depots, rather than intramuscularly, and thus, IM fat is deposited later than in sheep [46]. Fat deposition is also influenced by diet, sex or breed. For instance, meat purpose breeds, such as Hereford, have more SC and less abdominal fat at the same weight of total fat than milk purpose breeds, such as Holstein-Friesian. Moreover, late-maturing breeds have lower fat concentration than early-maturing breeds, at the same body weight [45]. 

IM fat is composed predominantly by PL, which have a high content of PUFA, and by TG, consisting mainly of SFA and MUFA. Furthermore, TG in IM tissue may vary considerably from 0.2% to 5%, but the PL content in the muscle is relatively constant, due to their role as structural constituents of the cells [38]. As indicated, in all animal species, including ruminants, most PUFA accumulate in PL in the muscles, and this fact limits high PUFA concentrations in meat. In addition, ruminants have smaller amounts of these fatty acids available for absorption into tissues, compared to monogastrics due to biohydrogenation of PUFA in the rumen, and the enrichment of ruminant products is less effective. However, the use of modified feeding can achieve useful levels of PUFA in meat animals, as described below.

## 3. Nutritional Strategies to Enhance the Fatty Acid Composition of Ruminant Meat by *n*-3 PUFA Addition

It has been shown that *n*-3 fatty acids may play a beneficial role in human health, especially due to their cardioprotective effects [1,2]. In order to obtain a product in compliance with the current nutritional guidelines in human nutrition, in the last decade, the use of sources rich in PUFA in ruminant feeding is a matter of interest, as a means towards modifying the composition of meat [47,48,49]. This involves the inclusion of a greater amount of omega-3 fatty acids in the diet, which entails a reduction in the ratio between omega-6 and omega-3 fatty acids (*n*-6/*n*-3).

The following section will focus on the effect of dietary *n*-3 PUFA on ruminant fatty acid composition, AT development and gene expression.

### 3.1. PUFA and Fatty Acid Composition of Ruminant Adipose Tissue

It is well known that PUFA composition of AT varies according to different factors such as age, muscle type, gender or breed, with diet being one of the main factors affecting it [50,51].

There is a wide variety of feed sources that are used to modify PUFA composition in meat obtained from ruminants. These ingredients can be different types of forages preserved differently, vegetable oils (such as sunflower, soja or canola), oilseeds (such as linseed and canola), and fish oils and marine algae [8]. This review will focus on the use of linseed and chia, rich in ALA, and on algae and fish oil as EPA and DHA source.

Linseed is one of the most studied seeds due to its high content in ALA (56%), in addition to oleic acid (17%) and LA (16.7%). Linseed is used to enrich meat in *n*-3 PUFA, and to promote the biosynthesis of long chain *n*-3 PUFA, such as EPA and DHA, considering that ALA is the precursor for endogenous synthesis of these fatty acids.

The effects of dietary supplementation of *n*-3 PUFA on the profile of the main categories of fatty acids in the IM and the SC AT of cattle and sheep are presented in Table 1 and Table 2, respectively. Results indicate that the inclusion of linseed, as oil or seed (whole, cracked or extruded), increases the content of ALA in the IM and the SC AT.

In cattle, the ALA content in the IM AT increased from 50.5% in high marbled Yanbian Yellow steers fed 8% whole linseed [52], up to a maximum of 550% in Holstein bulls fed 10% whole linseed [54], compared to those animals fed a control diet without *n*-3 PUFA supplementation. Similarly, in the SC AT, those authors observed a 50% and 690% increase in ALA with 8% cracked linseed [52] or 10% whole linseed supplementation [54], respectively.

In sheep, considering the results shown in Table 1, it could be inferred that it would be necessary to include a percentage of at least 3% of extruded linseed to obtain a significant increase in the content of ALA in the IM AT [61,66,78]. However, linseed levels below 3% increased the content of ALA in the SC AT [76]. The higher increase in ALA was observed in the IM (412%) and in the SC AT (850%) in Suffolk crossbred wether lambs fed 17.5% NaOH-treated linseed [64].

As a consequence, the increase in ALA in both the IM and the SC AT has a positive effect on the *n*-3 PUFA content, as well as on the *n*-6/*n*-3 ratio, that decreases significantly. This fact would indicate that both the SC and the IM AT from linseed-fed animals would be nutritionally healthier, due to the more favourable *n*-6 and *n*-3 fatty acids balance [79].

Another source rich in ALA used to increase PUFA levels in animal products is chia seed. This seed has the highest ALA content reported in plants so far (up to 64%) and, just as linseed, it contains also LA (around 19%) and oleic acid (9% approximately) [80]. The fatty acid profile found in lambs fed chia seed was similar to the one in lambs fed linseed, as shown in the research reported by Urrutia et al. [63], the only up to date study of the inclusion of chia seeds in AT of ruminants. It is worth mentioning that, even if the ALA content is higher in the chia seed compared to linseed, the increase in ALA content in the IM and the SC AT did not show significant differences. This fact might be due to a higher biohydrogenation of the ALA from the chia seed.

In cattle, the inclusion of linseed in the diet did not involve any changes in the LA content in the IM AT [52,53,54,55,56,57]. However, the inclusion of linseed in the diet showed inconsistent results in the SC AT. Li et al. [52] observed an increase in LA, whilst Fiorentini et al. [55] and Gonzalez et al. [56] reported a decrease in LA.

Concerning the results observed in sheep, only Bhatt et al. [58] reported an increase in LA when feeding lambs with linseed. On the contrary, other authors, such as Jeronimo et al. [60], Meale et al. [76] and Urrutia et al. [62], observed that the inclusion of linseed in the diet decreased LA in both the IM and the SC AT. Similarly, Urrutia et al. [63] observed a similar result when feeding lambs with chia seed, and Fan et al. [72] and Urrutia et al. [71] observed this when feeding lambs with algae.

Those results could be related to rumen biohydrogenation, which produces different intermediates, such as VA (C18:1*t*11) or C18:2*c*9*t*11 (CLA), among other fatty acids (see Section 2.1). CLA is originated by the biohydrogenation of LA and is the precursor of C18:1*t*11. In turn, C18:1*t*11 also has its origin in the biohydrogenation of ALA. In both the IM and the SC AT, part of dietary ALA could have escaped biohydrogenation and might have been incorporated to the IM and the SC AT, whereas another part could have undergone incomplete biohydrogenation causing the accumulation of C18:1*t*11 [81]. Some studies in lambs [62,63,64,71] observed a significant increase in C18:1*t*11 in the IM and the SC AT with the inclusion of linseed or chia seed in the diet, whereas other authors did not observe significant changes [61,65,66,74,76,77]. This result could be related to the different content and type of PUFA of dietary constituents used in these trials, which would result in varying degrees of biohydrogenation, and therefore a diverse content of biohydrogenation intermediate fatty acids in the tissues.

Regarding C18:2*c*9*t*11 (CLA) content, in cattle, an increase in this fatty acid was found by Li et al. [52], Gomez et al. [54] and Fiorentini et al. [55]. For instance, Gomez et al. [54] observed a 100% increase in the IM and the SC AT of Holstein bulls fed 10% whole linseed. In contrast, in sheep, the content of C18:2*c*9*t*11 (CLA) did not increase in the IM and the SC AT [61,62,63,65,66,68,70,71,74,76,77,78]. Only Bhatt et al. [58], Noci et al. [64] and Fan et al. [72] observed an increase in CLA. In addition, Suksombat et al. [53], who studied the effect of 3% linseed oil supplentation on Wagyu crossbred steers, observed a decrease in C18:2*c*9*t*11 (CLA) content. These authors argued that the decrease in C18:2*c*9*t*11 (CLA) may be due to a reduction in Δ9 desaturase activity, due to the dilution of C18:2*n*-6 by C18:*n*-3 in the diet, as C18:2*n*-6 is the direct precursor of C18:2*c*9*t*11, and hence C18:1:*t*11. They indicated that if CLA and *n*-3 PUFA have to be increased in order to improve the fatty acid profile of meat, a blend of C18:3*n*-3 and C18:2*n*-6 is required to supplement the diets of finishing steers. In this sense, in the above mentioned trials, in which an increase in CLA was observed in both the IM and the SC AT, the diets had a more balanced content of LA vs. ALA.

The major *cis*-MUFA in sheep meat is oleic acid (C18:1*c*9), which contributes 25–40% of the total fatty acid [82]. Several studies have found that in both the IM [61,62,63,64,65,70,71,73] and the SC AT [58,62,63,64,71] there is a decrease in the content of oleic acid with the inclusion of linseed (crushed, extruded or oil), chia seed or algae in lamb’s diets. Δ9 desaturase desaturase (*SCD* gene) plays an important role in converting C18:1*t*11 into C18:2*c*9*t*11 (CLA), and it is also responsible for converting SFA to MUFA, primarily stearic acid (C18:0) into oleic acid (C18:1*c*9). It is plausible that the observed decrease in oleic acid could be the result of the inhibition of *SCD* by dietary PUFA (see Section 3.3).

In contrast to the results observed in most cases, Li et al. [52] found an increase in the percentages of C18:1*c*9 and C18:2*c*9*t*11 in both the IM and the SC AT, and also C18:2*n*-6 (LA) in the SC AT of Yanbian Yellow steers fed 8% whole and cracked linseed. This could be due to the fact that the Yanbian Yellow is a highly marbled breed, closely related to Korean Hanwoo cattle [52], and could have higher fatty acid deposition capacity. In this sense, Zembayashi et al. [83] reported that Japanese Black, another highly marbled breed, has a genetic predisposition for synthesis and deposition of C18:1 or MUFA in SC and IM neutral lipids. Insausti et al. [84] also reported that Morucha, a Spanish local beef breed, could have a genetic predisposition for depositing MUFA, which might be related to its high IM fat content.

Concerning the amount of EPA, some authors [48,61,62,66,78] did not observe significant changes in EPA content with linseed addition. However, results from different trials carried out with beef [53,54,55,56] and lambs [59,60,63,64,65,68,71] fed linseed showed a significant increase in this fatty acid. In cattle, Suksombat et al. [53] reported a 238% increase in EPA from 3% linseed oil supplementation, compared to the control in the IM AT of Wagyu crossbred steers. Likewise, Fiorentini et al. [55] observed that 4.5% linseed oil inclusion increased the content of this fatty acid in the SC AT of Nellore steers (+ 325%). In sheep, Noci et al. [64] found an increase in the content of EPA in the IM (329%) and the SC AT (600%) in Sulfflok fed 17.9% NaOH-treated linseed. If algae or fish oil is also added to the diet, then the increase in EPA is more pronounced. For example, up to a 959% increase in EPA was observed in Manchega lambs fed fish oil [68].

As far as DHA content is concerned, linseed inclusion in the diet did not have a significant effect on the IM and the SC AT (Table 1 and Table 2), even if its precursor, ALA, increased when including linseed in the diet. This result could be related to the limited desaturation and elongation of from ALA to *n*-3 LCPUFA, suggesting that a metabolic pathway “blockage” had occured at the docosapentaenoic acid (DPA) level, as it was also suggested by other authors [8,85]. Only in those works where linseed oil or NaOH-treated linseed were used, an increase in DHA was observed in the IM fat depot. This linseed form might have limited ruminal biohydrogenation and, thus, the amount of ALA that reached the AT was higher.

Consequently, among the different feeding strategies used to enrich meat in *n*-3 PUFA and *n*-3 LCPUFA (Table 1), the inclusion of algae or fish oil led to the highest increase in DHA content in the IM and the SC AT. For instance, meat from lambs fed with a diet containing 3.9% algae [71] or 3.3% fish oil [68] showed an increase of 1000% in DHA content compared to lambs fed a diet not containing these *n*-3 PUFA sources. Thus, the use of *n*-3 LCPUFA rich sources of marine origin (fish oil and algae) is the most effective feeding strategy to increase the EPA and DHA contents in meat.

In order to help consumers make healthier choices, the European authorities [86] adopted the Regulation number 116/2010 about the nutrition claims identifying foods being source of PUFA fatty acids. As stated in this Regulation, a claim that a food is “Source of omega-3 fatty acids” may only be made when the product contains at least 300 mg of ALA/100 g, or at least 40 mg of the sum of EPA+DHA/100 g. A claim that a food has “High content of omega-3 fatty acids” may only be made when the product contains at least 600 mg of ALA/100 g, or at least 80 mg of EPA+DHA/100 g. Therefore, the results shown in both cattle and sheep summarized in Table 1 and Table 2 highlight that animals fed with algae or fish oil reach the levels required to be labeled as a “Source of omega-3 fatty acids”. For example, in the work of Urrutia et al. [71] and Ponnampalam et al. [48] in meat from lambs fed with linseed and algae, the sum of EPA and DHA had approximate values of 56.7 and 66.8 mg/100 g, respectively. Hopkins et al. [73] and Meale et al. [77] observed values of EPA and DHA of 140 mg/100 g and 147.7 mg/100 g in meat of lambs fed 2% and 3% algae, respectively, which would allow labelling this meat as “Food high in omega-3 fatty acids”.

It is worth mentioning that the variation of fatty acid composition of meat can affect its quality, as high PUFA levels may affect in meat flavour, due to their susceptibility to oxidation [87]. Therefore, it is important to take into account the potential implications of the modification of fatty acid composition in the lipid stability and in meat quality.

### 3.2. PUFA and Adipose Tissue Development

#### 3.2.1. Animal Growth and Carcass Characteristics

Fat supplements to ruminant animals in recent years are been used with the goal of increasing the levels of PUFA in products ultimately intented for human consumption. But it is important to take into account that several factors, such as level and type of supplemental fat can affect animal growth and weight gain [88]. Then, different studies investigating the effect of linseed, as *n*-3 PUFA source (ALA), on the animal growth and carcass characteristics, were carried out. Overall, the majority of the results showed that linseed addition did not affect animal growth and carcass characteristics. Wachira et al. [89] observed that lambs fed 10.5% linseed had similar dry matter intake (DMI), daily weight gain, final live weight, carcass weight and SC fat percentage. Also, lamb growth and carcass parameters were not affected by adding 9% [61] and 10% [62,63,71] extruded linseed and 8.5% NaOH-treated linseed [90]. In addition, Noci et al. [64] did not show significant differences between lambs fed a concentrate-based diet and those fed 17.9% linseed or 6% linseed oil, but feed intake and carcass weight was higher, and perirenal fat weight tended to be higher for lambs offered the linseed oil than for the NaOH-treated linseed. In contrast to the former results, Noci et al. [64] reported that 6% linseed oil supplementation increased slaugther weight, carcass weight and fat percentage of chump and shoulder, compared to the control group fed dehydrated lucerne with sunflower oil. In cattle, the results indicated that animal performance and carcass characteristics were not affected by linseed supplementation (cracked, whole seed or oil) [52,53,54,56,57]. For example, Albertí et al. [57] reported that adding linseed to the diet of young Holstein bulls did not exert any significant effect on the 10th rib tissue composition (SC and intermuscular fat).

Regarding marine algae supplementation to ruminant diets as source of *n*-3 LCPUFA (EPA and DHA), reduced DMI and average daily gain of growing lambs fed 2% algae [70] and 3.89% algae with 5% linseed [71] was reported, but carcass characteristics were not affected in the latter case. Also, Burnett et al. [91] indicated that 1.8% algae supplementation to growing lambs decreased DMI, although growth rate and carcass weight were not affected. In this experiment, lambs offered both linseed flakes and algae supplement had higher back-fat thickness than lambs offered a pelleted annual pasture hay diet without fat suplementation. In contrast, de la Fuente-Vázquez et al. [68] did not observe a decrease in feed intake or average daily gain of algae and linseed (4% and 10.5%, respectively)-fed lambs. Likewise, Hopkins et al. [73] and Meale et al. [77] observed similar results in lambs fed with algae.

On the other hand, the majority of studies investigating fish oil supplementation have reported decreased feed intake and growth rate in lambs [68,89,92] and feed intake in steers [93] with up to 3.6% fish oil inclusion. These results could be caused by reduced palatability of diets [92,94], which would be accompanied by a decrease in microbial growth in the rumen and shifts in ruminal fermentation [95], in addition to fiber degradation [89,92]. Moreover, an increase in carcass fat scores was reported by Wachira et al. [89] in lambs fed 3.6% fish oil, and by Demirel et al. [90] in lambs fed 1.5% linseed and 1.5% fish oil.

#### 3.2.2. Cellularity (Fat Cell Size and Number)

Hyperplasia (increase in cell number) and hypertrophy (increase in the volume of cells) are the two mechanisms responsible for AT growth. Proliferation of preadipocytes and their subsequent differentiation (hyperplasia) takes place mainly during the animal foetus and postnatal period. Hypertrophy also take place during this same period, whereby adipocyte’s volume increases by the accumulation of lipids. These lipids are stored in the form of TG, that come from the esterification of G3PDH, which needs glucose and fatty acids (from the diet or de novo) to be synthesised [96,97,98,99].

After birth and at an early postnatal period, AT growth is primarily due to hypertrophy of existing adipocytes, as well as to the activity of lipogenic enzymes, namely FAS, ACC, G3PDH, G6PHD and ICDH [100]. However, the AT size is not necessarily determined by the early age hyperplasia, inasmuch as adipocytes can stimulate adipogenesis when a given percentage of adipocytes have reached their maximum volume, thereby inducing an increase in hyperplasia and/or promoting lipid accumulation by preadipocytes, which had been quiescent until then [6,97,101].

Several factors, such as sex, breed, age, physical condition, diet or anatomical location of the adipose depots can influence both hyperplasia and hypertrophy [102]. The current section will focus on the effect of diet on AT growth. At the cellular level, the quantification of the size and number of fat cells can be used for the characterization of the AT growth process. Nevertheless, studies in ruminants to address dietary effects on the dynamic process of AT growth measuring cell-size distributions are scarce.

**Figure 5:**
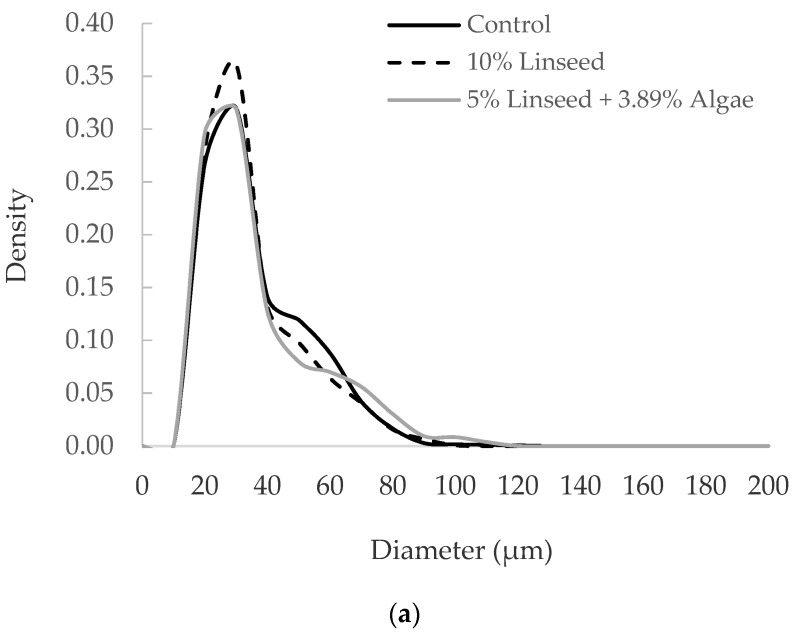

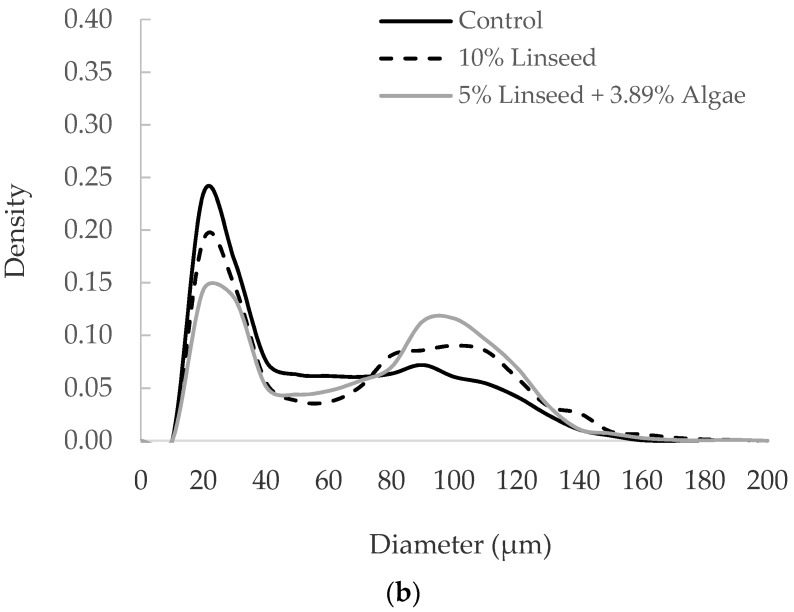
Effect of addition of linseed or linseed and algae to the diet on the adipocyte size distribution of lambs. Data reanalyzed from Urrutia et al. [71] (see Supplementary Material 1–3). (**a**) Analysis of intramuscular adipocyte size distribution of Navarra breed lambs; (**b**) analysis of subcutaneous adipocyte size distribution of Navarra breed lambs.

In sheep, Urrutia et al. [71] studied the effect of linseed or linseed with algae supplementation on adipocyte size distribution of the IM and the SC AT in Navarra breed light lambs. As shown in Figure 5a, the size distribution of adipocytes from the IM AT in the control group and two experimental groups was unimodal, with the largest proportion of adipocyte cells within the range of 20–30 μm diameter in all the groups. Likewise, the results from a study with cattle [103] indicated that adipocyte size distribution of the IM AT in Holstein young bulls were unimodal, with a significant proportion of small adipocytes. The higher density of small adipocytes observed in sheep and cattle could indicate that the development of this depot may be mainly due to hyperplasia or cell proliferation, which corresponds to early phases of fat accretion [96,104]. From these data, it appears that the IM AT behaves differently from other AT that normally develop early, such as the SC, visceral or perirenal fat. The IM AT can be considered as a later maturing tissue in cellularity development, and it starts to accumulate fat in the late stages of growth [104,105,106,107]. This fact could also be the result of the constriction caused by muscle fibers on the adipocytes. Also, IM adipocytes are reported to have a lower lipogenic, lipolytic, fatty acid oxidative, fatty acid transport and(or) energy transfer capacities, compared to larger adipocytes isolated from the SC or perirenal depots of growing animals [71,108,109].

In the SC AT in lambs, in contrast to the results observed in the IM AT, the adipocyte size distributions in the three dietary groups were bimodal, indicative of two-cell populations (Figure 5b). Bimodality in adipocyte distribution might reveal the simultaneous occurrence of both hyperplasia, represented by the population of small adipocytes, and the hypertrophy, represented by the large adipocyte population [71]. In cattle, although Albertí et al. [103] indicated that the SC adipocyte size distributions in both the control and 10% linseed-fed Holstein young bulls follow a normal distribution, other authors, such as Soret et al. [104] and Martínez del Pino et al. [110], observed bimodal adipocyte size distribution in the SC AT of young Pirenaica bulls fed a concentrate-based diet, and Schoonmaker et al. [111] also observed this in 10-month-old Holstein steers.

Not only did the IM and the SC AT reveal distinct adipocyte size distributions, but also diverse pattern of fatty acids. Thus, the *longissimus* muscle had higher proportions of PUFA, *n*-6 PUFA and LA. In lambs, Bas et al. [61] reported a percentage of 6.8% of PUFA, 5.2% of *n*-6 PUFA and 4.1% of LA in the IM AT, whereas in the SC AT, the percentages were 4.8%, 2.8% and 2.6% for PUFA, *n*-6 PUFA and LA, respectively. These results could be related to the fact that the SC AT is characterized by a higher adipocyte size (above 100 µm) than the IM AT (25–50 µm) [104,110], and by a high proportion of TG (up to 90%). However, the PUFA content, especially in EPA and DHA, is low, because PL make up only a small proportion of the total [47,112,113]. In contrast, the IM AT is characterized by a smaller adipocytes size compared to the SC AT [71,103]. Small cells provide a greater density of cellular membranes, which is why the IM AT is mainly formed by PL, which have a high PUFA content [114]. In this regard, Noci et al. [64] and Jeronimo et al. [60] determined the total neutral and polar lipids of *longissimus* muscle. The published data showed that LA, ALA, EPA and DHA were at much higher proportions in PL than in neutral lipids (LA, ALA, EPA, DHA were on average 6, 3–4, 35–36, 12–15 times higher in polar lipids, respectively) whilst C18:1*t*11 and CLA were preferably deposited in the neutral lipid (2–3 times higher in neutral lipids).

With reference to the dietary PUFA effect in the IM AT, Urrutia et al. [71] indicated that increased consumption of ALA and/or EPA and DHA did not exert a significant effect on adipocyte number and mean adipocyte diameter of lambs fed 10% linseed or 5% linseed plus 3.89% algae, compared to non-PUFA supplemented lambs (55.5, 64.5 and 67.6 µm, respectively). Moreover, there were no notable differences in the relative frequencies among all adipocyte classes (<30 µm, 30–60 µm and >60 µm) (Figure 5a). They reported that these results were consistent with the similar percentage of the IM fat among three dietary groups. Similarly, in cattle, Albertí et al. [103] addressed that the supplementation of young bulls with a high ALA diet (10% linseed) during fattening produced no difference in adipocyte size and number in the IM AT, compared to the control animals fed a diet containing the same amount of fat, but devoid of *n*-3 PUFA.

**Figure 6:**
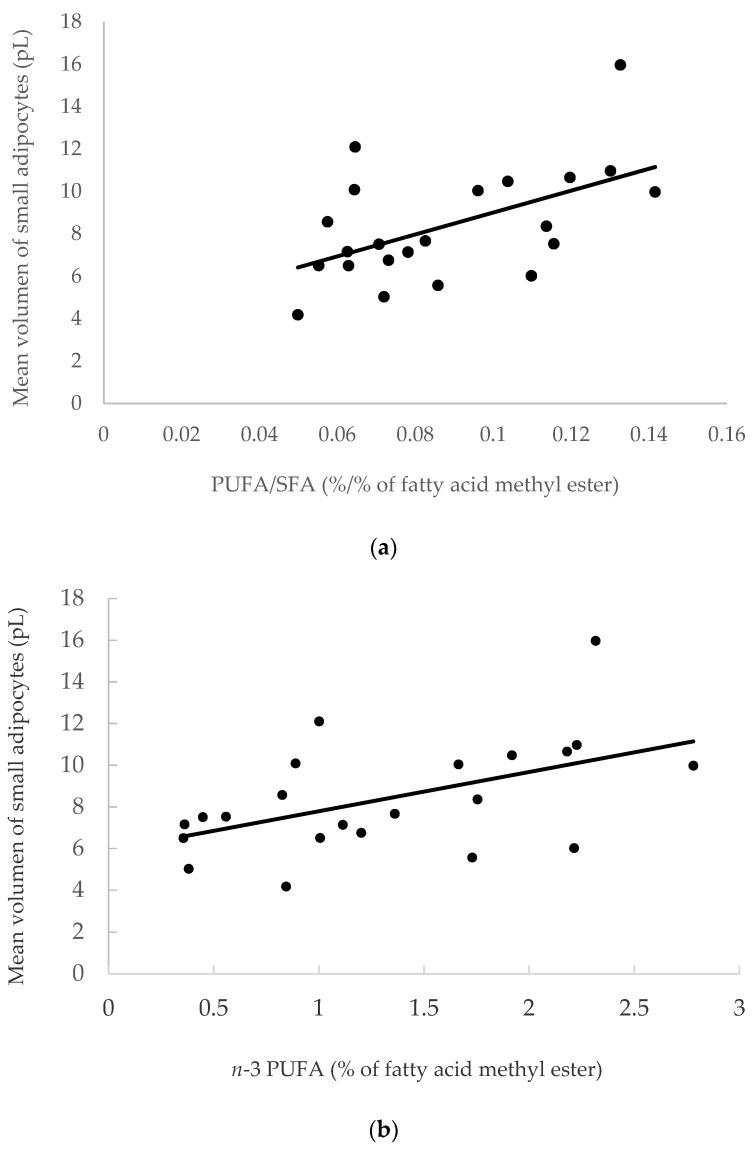
**(a)** Positive correlation between PUFA/saturated fatty acids (SFA) and mean volume of small adipocytes in the subcutaneous (SC) adipose tissue (AT) (*r* = 0.539; *p* = 0.0097); (**b**) positive correlation between *n*-3 PUFA and mean volume of small adipocytes in the SC AT (*r* = 0.512; *p* = 0.015).

In the SC AT, Urrutia et al. [71] reported that *n*-3 PUFA diet supplementation, particularly with linseed (ALA) and algae (EPA and DHA), may stimulate adipocyte hypertrophy in lambs, as shown by the high frequency of 90–120 µm adipocytes observed in lambs fed *n*-3 PUFA supplemented diet, compared to control group lambs (Figure 5b). Using the data from cited work (Table S1), the relationship between fat cell size and number and fatty acid composition of the SC AT calculating Pearson´s correlation coefficients were examined in bimodal distributions. In agreement with those results, a significant positive correlation between PUFA/SFA, *n*-3 PUFA and ALA contents with the volume of small adipocytes (*r* = 0.539, *p* = 0.0097; *r* = 0.512, *p* = 0.015; *r* = 0.537, *p* = 0.0099, respectively) was found. The correlation between PUFA/SFA and *n*-3 PUFA with the volume of small adipocytes in the SC AT of bimodal distributions is shown in Figure 6.

Likewise, the *n*-6/*n*-3 ratio was positively related to the number of small adipocytes (*r* = 0.631, *p* = 0.0016) and the ratio of the number of small/large adipocytes (*r* = 0.627, *p* = 0.0018), and negatively related to the percentage of adipocyte above the nadir (midway point between two cell populations) (*r* = -0.574, *p* = 0.0053). Overall, these data would indicate that the linseed and linseed plus algae supplemented diets, which resulted in higher *n*-3 PUFA and ALA contents and lower *n*-6/*n*-3 ratio in the SC AT, would have stimulated the hypertrophy of small adipocytes, and thus, the proportion of larger adipocytes with 90–120 µm of diameter would have increased.

Urrutia et al. [71] also indicated that the higher hyperthrophy of small adipocytes would be the result of the activation of TG synthesis by dietary ALA, EPA and DHA, which would be mediated by *PPARG* and *CEBPA*. In this sense, an increase in the expression of transcription factor *PPARG* and *CEBPA* (see Section 3.3), and the activity of the enzyme G3PDH involved in TG synthesis was reported (Table S2). As an adipogenic factor, *PPARG* plays multifaceted roles, and it is able to regulate the TG synthesis and the fatty acid de novo formation, and it is also a key factor regulating the process of differentiation of fat cells. In contrast to the results reported in sheep, in cattle, Albertí et al. [103] did not find any dietary ALA effect on adipocyte size, number and fat cell distributions and lipogenic enzyme activity.

Todorčević and Hodson [115] reviewed the available data of EPA and DHA effects on white AT in rodents, humans and in vitro cellular cultures. In contrast to what the limited data in ruminants show, the majority of works on AT and mice models have found that these fatty acids may limit lipid accumulation in AT, as they reported lower fad pad mass and adipocyte number and size with EPA and DHA supplementation. Moreover, the authors also mentioned that works from in vitro cellular studies have found that EPA and DHA inhibit, promote or have no effect on the differentiation of pre-adipocytes. For instance, Murali et al. [116] observed that incubating 3T3-L1 with EPA and DHA induced the differentiation process (increased lipid droplets), and increased the expression of *PPARG*, *CEPBA* and *FABP4*. Conversely, Ferreira et al. [117] recently found that 3T3-L1 adipocyte groups treated with EPA, DHA and EPA+DHA showed smaller adipocytes in comparison to non-treated control group and decreased *PPARG* and *CEBPA* gene expression. In humans, some studies suggested that the increased consumption of EPA and DHA may decrease body fat [115], whereas others found no evidence to support an anti-obesity role of *n*-3 PUFA [118]. Taken all this together, and considering the divergent findings, the effect of ALA, EPA and DHA fatty acids in modulating AT cellularity remains to be elucidated.

### 3.3. PUFA and Gene Expression Related to Adipogenesis and Lipid Metabolism

Dietary type and amount of fat can modulate the expression of genes responsible to encode enzymes involved in fat tissue deposition by affecting the level and the profile of fatty acids in meat from ruminants [12,119]. It has been reported that the effects of PUFA on AT are related to changes in the expression of genes involved in lipid metabolism in adipocytes [5]. In this sense, although there have been no transcriptomic studies investigating the effect of PUFA on ruminant AT, in the bovine mammary gland, the analysis of the transcriptome by RNA sequencing revealed differential expression of 1006 genes when Holstein cows were fed 5% linseed, indicating that this diet downregulated genes involved in the fatty acid/lipid synthesis and lipid metabolism pathways [120].

**Figure 7:**
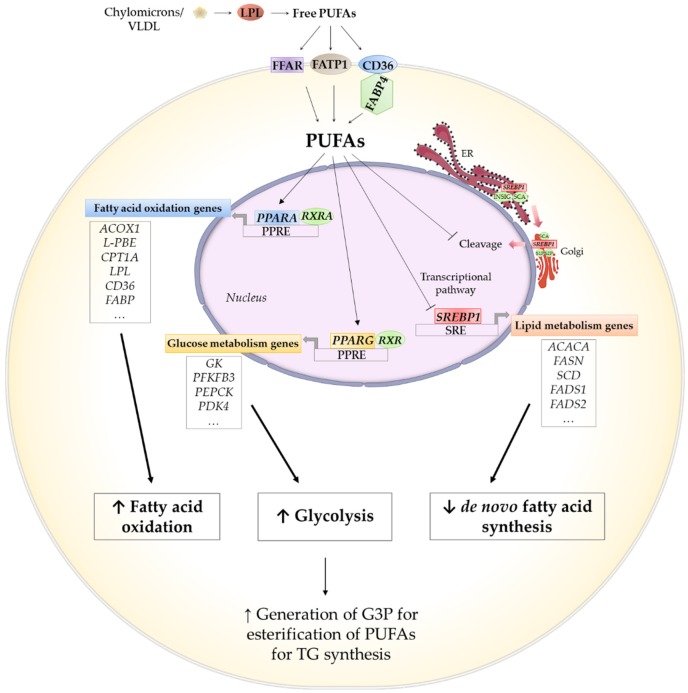
Diagram illustrating potential mechanism of gene regulation by polyunsaturated fatty acid (PUFA). Source: own elaboration. PUFA act as ligands for *peroxisome proliferator–activated receptors* (*PPAR*). Activation of *PPAR alpha (PPARA)* by PUFA promotes the transcription of enzymes involved fatty acid oxidation and fatty acid transport. Activation of *PPARG* is likely to increase glucose metabolism for generation of glycerol-3-phosphate (G3P) for esterification of long chain PUFA for triacylglyceride (TG) synthesis. PUFA inhibit the proteolytic processing and transcription of *sterol regulatory element-binding protein* (*SREBP*) *1*. Abbreviations: *ACACA* = *acetyl*-*CoA carboxylase*; *ACOX1* = *acyl-CoA oxidase*; *CD36* = *fatty acid translocase*; *CPT1A* = *carnitine palmitoyltransferase 1A*; *FABP4* = *fatty acid-binding protein 4*; *FADS1* = *fatty acid desaturase 1; FADS2* = *fatty acid desaturase 2; FASN* = *fatty acid synthase*; *FATP1* = *fatty acid transport protein*; *FFAR* = *free fatty acid receptor*; *GK* = *glucokinase*; *L-PBE* = *peroxisomal enoyl-CoA hydratase*; *LPL* = *lipoprotein lipase*; *PDK4* = *pyruvate dehydrogenase kinase 4*; *PEPCK* = *phosphoenolpyruvate carboxykinase*; *PFKFB3* = *6-phosphofurcto-2-kinase/fructose-2,6-bisphosphatase 3;* PPRE = peroxisome proliferator response element; *RXRA* = *retinoid X receptor alpha*; *SCD* = *stearoyl-CoA desaturase*; SRE = sterol regulatory element; VLDL= very low density lipoprotein.

It has been reported that fatty acid, in particular PUFA, exert their biological effect through regulating the activity of numerous transcription factors, including the carbohydrate response element binding protein (ChREBP) and sterol regulatory element-binding proteins (SREBPs) [121,122]. Also some early studies, both in vivo and in vitro, showed that PUFA are the best endogenous or natural activators of peroxisome proliferator-activated receptors (PPARs), with *PPAR alpha (PPARA)* being activated by fatty acids [123] (Figure 7).

PPARs are a family of transcription factors which belong to the superfamily of steroid receptors. These nuclear receptors bind to specific target sequences in the promoter regions of numerous genes, and control the transcription of specific genes in response to nutrient signals. The PPARs perhaps compose the best recognized sensor system for fatty acids [124]. The isoform *PPARA* is highly expressed in metabollically active tissues, such as the liver, heart, skeletal muscle or brown AT [125]. In mammals, *PPARA* is activated by fatty acids or their derivatives, and can stimulate the expression of genes related to fatty acid metabolism, including their transport through cell membranes, intracellular union, peroxisomal and mitochondrial β-oxidation, and also LCPUFA biosynthesis [122,126]. The works studing the effect of PUFA on *PPARA* expression in ruminants are scarce. Kronberg et al. [127], did not observe significant differences in *PPARA* expression in the *longissimus dorsi* muscle of Angus steers fed linseed. In contrast, Gruffat et al. [128], observed an increase in the mRNA levels of *PPARA* in the Angus and Blonde d´Aquitaine breeds, in the same muscle of the bulls fed linseed, suggesting that PUFA would increase fatty acid oxidation through this transcription factor, which has already been described in humans and rodents and other tissues such as liver [129,130,131,132]. Although *PPARA* expression did not increase in Limousin bulls as in the other two breeds, an increase was found in the expression of nuclear receptor *RXRA*, which is required for *PPARA* transcriptional activity on fatty acid oxidation genes such as *fatty acyl-CoA oxidase 1* (*ACOX1*) or *peroxisomal enoyl-CoA hydratase* (*L-PBE*) [131]. Indeed, the former genes increased in linseed fed bulls.

Only one of the isoform of PPAR family, *PPARγ* or *PPARG*, is highly expressed in AT, and it is a master regulator of adipocyte differentiation, along with *CEPBA* [133]. *PPARG* is expressed during the late stage of adipocyte differentiation and remains abundantly expressed in differentiated adipocytes, regulating the expression of genes involved in lipid metabolism including fatty acid uptake and transport (*LPL*, *CD36*, *FATP1* or *FAPB4*) or lipogenesis (*ACACA*, *FASN, ME* or *SCD*) [134]. In addition, Nakamura et al. [134] suggested that *PPARG* targets a variety of genes involved in glucose metabolim and, as the profile of *PPARG* targets indicate, the main effect of *PPARG* activation is not an induction of de novo lipogenesis, but the increased generation of G3P for PUFA esterification, and subsequently for TG shyntesis.

Some authors indicated no effect of linseed, algae or fish oil on the expression of *PPARG*, neither in cattle nor in sheep in the IM [71] or the SC AT [75,135,136,137] (Table 3). In contrast, Li et al. [52] and Kronberg et al. [127] reported that the expression of *PPARG* significantly increased in the *longissimus* muscle of linseed-fed steers. Furthermore, Fan et al. [72] found an increase in the expression of *PPARG* in the same muscle of lambs supplemented with 3% algae. In the SC AT, Urrutia et al. [71] also observed an upregulation of this transcription factor by linseed or linseed plus algae supplementation in lambs, along with increased *CEBPA* mRNA levels. Additionaly, in goats, Ebrahimi et al. [138,139] reported an increase in *PPARG* expression in the SC AT and the *semitendinosus* muscle in 1.3% linseed oil-fed Boer goats. These results would support previously cited hypothesis by Nakamura et al. [134], suggesting that *PPARG* activation is not intended to induce de novo lipogenesis, as the majority of the works indicated that PUFA addition to the diet downregulated the expression of the main genes involved in de novo lipogenesis, mainly *ACACA* but also *FASN,* which encodes the enzymes ACC and FAS, respectively (Table 3).

On the other hand, as suggested by Li et al. [52], PUFA depressed de novo fatty acid synthesis and promoted the uptake of dietary fatty acids. They observed that 8% linseed supplementation increased the expression of *LPL* in the IM AT of steers. In line with this, Ladeira et al. [25] indicated that the expression of genes involved in fatty acid transport into the cell, *CD36*, *FFAR2*, *FFAR4* and *FFAR1*, increased in the IM AT of lambs fed linseed oil, compared to the control diet lambs. In addition, an increase in the expression of *LPL* was also reported in the SC AT of 1% fish oil-fed Holstein cows by Thering et al. [135], and in the SC and the IM AT of 5% linseed-fed lambs by Urrutia et al. [62]. Other authors, in contrast, found that PUFA either have no effect or inhibit *LPL* expression. For instance, mRNA level of *LPL* was reduced when 8% linseed was added to the diet in the SC AT of steers [75]. In sheep, a similar result was reported, with 10% chia seed supplementation in the IM AT and with 5% linseed plus 3.89% algae in both the SC and the IM AT. Overall, the data would indicate that dietary PUFA exert a regulatory effect on *LPL* expression in the SC and the IM AT, and it seems to respond differently, depending on the percentage and type of fatty acids included in the diet, which was also previosly indicated by other authors [146,147]. Elucidating this question will require further research into these aspects.

Regarding fatty acid desaturation, *SCD* gene (encodes Δ9 desaturase) plays an important role in converting C18:1*t*11 (VA) into C18:1*c*9*t*11 (CLA), and it is also responsible for converting SFA to MUFA, primarily stearic acid (C18:0) to oleic acid (C18:1c*9*). *SCD* is modulated by fatty acids and cholesterol [142,148,149]. The majority of the published works have reported that diets rich in ALA, EPA and/or DHA downregulated *SCD* expression (Table 3). The regulation of *SCD* by fatty acids seems to occur at the levels of both transcriptional and enzyme activity [150]. In cattle, the mRNA level of *SCD* decreased in the IM AT of 8% linseed [52] and up to 2% fish oil [142]-fed animals. Moreover, a similar result was found in the SC AT of 8% linseed-fed young bulls. Likewise, Hiller et al. [140] reported that grass silage (*n*-3 PUFA-based diet) significantly reduced *SCD* expression in the IM AT, compared to the maize silage-based diet (*n*-6-based diet). In sheep, a decrease in *SCD* expression was reported in the IM and the SC AT of linseed oil [25], linseed [62] or algae [71,72]-fed lambs. Ebrahimi et al. [145] also observed the downregulation of *SCD* the *longissimus dorsi* muscle of goats fed a diet containing 1.3% linseed oil.

Other authors indicated that as a result of the inhibition of *SCD* by dietary PUFA, the content of C18:2*c*9*t*11 (CLA) did not increase, or even decreased in some cases in animals fed linseed or algae, despite the higher availability of its precursor VA [62,63,71,75]. Similarly, the content of oleic acid did not increase [62,72,75] or even decreased [63,71]. It was suggested that the regulation of *SCD* in response to dietary PUFA would contribute to the cells homeoviscous adaptation mechanism, whereby they preserve the fluidity of their membranes, maintainig the constancy of unsaturation degree of C18 fatty acids to ensure cell membrane functions [60,62,151]. Ballweg and Ernst [152] stated that one of the key factors determining membrane fluidity and phase behaviour is the proportion of saturated and unsaturated acyl chains in membrane lipids, with the Δ9 desaturase (encoded by *SCD* gene) being crucial in mantaining membrane fluidity.

The synthesis of LCPUFA is controled principally by the enzymes encoded by *FADS1* and *FADS2*, and its dietary regulation by PUFA is still poorly understood in AT [153]. In cattle, a diet rich in ALA, EPA and/or DHA did not affect the expression of *FADS1* and *FADS2* in the IM [140] and the SC AT [135,137,140]. On the other hand, in sheep, contradictory results have been reported about the effect of PUFA on the expression of the former genes. In the SC AT, it has been indicated that there was no effect of linseed or linseed plus algae on *FADS1* and *FADS2* expression in lambs [62,71], which is in line with the results of Rodrigues et al. [144] and Coleman et al. [136] in lambs fed EPA and DHA-rich diets. In contrast, in the IM AT, dietary ALA, EPA and/or DHA exert a more pronounced effect. Urrutia et al. [71] found that both 10% linseed or 5% linseed plus 3.89% algae significantly reduced the expression of *FADS1* and *FADS2* in light lambs. Likewise, similar response was observed in 10% chia seed-fed light lambs [63]. In addition, Fan et al. [72] indicated that 3% algae supplementation reduced the *FADS2* mRNA level. In accordance with these results, dietary supplementation of ALA, the precursor of LCPUFA, did not alter the levels of the final product DHA (Section 3.1). Ralston et al. [153] proposed that the endogenous synthesis of DHA in adipocytes is tightly regulated, considering the low rate conversion between ALA and DHA. Thus, the above gene expression results suggest that the transcriptional regulation of *FADS1* and *FADS2*, mainly in the IM AT, would be one of the mechanisms to regulate LCPUFA synthesis.

SREBPs are important transcription factors that regulate the metabolism of cholesterol and fatty acids, activating genes required for de novo lipogenesis, such as *ACACA*, *FASN* and *SCD* [154]. SREBPs are central for maintaining cellular lipid homeostasis [132]. Studies over the past decade showed that PUFA reduced *SREBP1* mRNA levels and inhibit proteolytic processing of *SREBP1* [124]. In cattle, the expression of *SREBP1* was downregulated in the IM AT of steers fed 8% cracked linseed [52] and 2% fish oil [142], and in the SC AT of bulls fed grass silage [141]. Similarly, Waters et al. [142] found a positive relationship between *SREBP1* and *SCD* gene expression, with the expression of both genes being negatively correlated to the tissue concentrations of *n*-3 PUFA.

In sheep, Fan et al. [72] also observed a reduction in mRNA levels of *SREBP1* in lambs fed 3% algae. According to these results, it is likely that dietary PUFA decreased the expression of genes involved in de novo fatty acid synthesis, *ACACA* and *FASN*, by a repression of the *SREBP1* transcription factor [25,122]). In contrast, other authors did not observe changes in the mRNA levels of *SREBP1* in the IM and the SC AT when animals were fed high-PUFA diets [25,71,135,137,155]. Different mechanisms, whereby PUFA regulate lipogenesis through *SREBP1*, have been proposed [122]. PUFA can inhibit *SREBP1* maturation in the nucleus or lower the stability of *SREBP1* mRNA, as well as its expression, so this could be the reason for the different results observed about *n*-3 PUFA on *SREBP1* expression.

Overall, it seems that PUFA would regulate the transcription of nuclear receptor such as *PPARA*, *PPARG* and *SREBP1* involved in the control of lipid homeostasis and fatty acid uptake, changing the adipocyte metabolism towards the activation of fatty acid oxidation and downregulation of de novo fatty acid synthesis.

## 4. Conclusions

This review summarizes different strategies based on dietary *n*-3 PUFA supplementation to enhance the fatty acid profile of ruminant meat intended ultimately for human consumption. Some feed sources of *n*-3 PUFA have a potential benefit in changing the fatty acid profile of meat by adding those ingredients to animal diets, such as linseed, chia seed, fish oil or marine algae, although not all of them have the same effects. Whilst linseed supplementation leads to an increase in ALA and EPA, but not in DHA in the IM and the SCAT, fish oil and algae inclusion raise the DHA levels.

The addition of PUFA to the diet can alter AT cellularity and consequently affect its metabolic activity, although those effects are not totally elucidated, as both anti- and pro-adipogenic effects have been reported, and thus, more studies would be needed. Additionally, dietary PUFA modulate the expression of genes involved in lipid metabolism, controlling the fatty acid uptake and oxidation and downregulating de novo fatty acid synthesis, although the exact mechanism is still unclear.

As reviewed, adipogenesis and metabolism of AT are complex processes regulated by a wide range of factors, many of them well-known, but most of them interdependent. This makes it difficult to study their main effects, which offer some contradictory results when using similar feeding strategies. Thus, it would be desirable to consider their interaction in future research.

## Figures and Tables

**Table 1:** Results of different studies of the effect of dietary inclusion of *n*-3 long chain polyunsaturated fatty acid (PUFA)-rich sources on the fatty acid composition of intramuscular fat in ruminants. Effects are shown as relative variation with respect to the control diet and expressed in percentage.

	Inclusion Level, Live Weight ^1^	Unit	C18:3*n*-3 ^2^	C20:5*n*-3	C22:6*n*-3	C18:2*n*-6	C18:1*t*11	C18:1*c*9	C18:2*c*9*t*11	*n*-6/*n*-3	Reference
**Cattle**											
Whole linseed	8%, 500–700	% total FA	52.5 *	50.0	0.0	6.7	24.1	13.8 *	54.1 *	-	[52]
Cracked linseed	8%, 500–700	% total FA	78.7 *	50.0	0.0	10.3	21.5	14.7 *	47.7 *	-	[52]
Linseed oil	3%, 640–718	% total FA	231.3 *	237.5 *	195.7 *	−40.8	−20.3 *	1.6	−41.0 *	−79.2 *	[53]
Whole linseed	10%, 240–458	% total FA	550.0 *	140.0 *	0.0	−17.6	24.2 ^4^	8.5	100.0 *	−77.8 *	[54]
Linseed oil	4.5%, 419, −(90 d ^3^)	% total FA	106.7 *	86.7	-	−14.6	-	−3.9	81.1 *	−56.6 *	[55]
Linseed oil	4.5%, −344	% total FA	112.0 *	51.7 *	0.0	−4.9	−23.0	3.7	−13.0	−42.3 *	[56]
Whole linseed	5%, 278–440	% total FA	162.7 *	35.0	-33.3	−26.4	2.6	19.6	-	−41.4 *	[57]
**Sheep**											
Crushed linseed	10%, −38	% total FA	171.0 *	-	78.9	20.1 *	-	2.4	73.7 *	−46.7 *	[58]
Extruded linseed	15%, 21–30	% total FA	207.7 *	288.9 *	-	−30.9	-	3.0	-	−86.4 *	[59]
Extruded linseed	30%, 21–30	% total FA	223.1 *	261.1 *	-	−2.5	-	−6.1	-	−79.0 *	[59]
Linseed oil	6%, 21–30	% total FA	228.0 *	163.2 *	42.9 *	−45.6 *	−17.8 *	2.3	-	−77.3 *	[60]
Extruded linseed	3%, 24–38	% total FA	97.7 *	-6.3	-16.7	−15.6	−30.0	−6.8 *	−58.3	−45.6 *	[61]
Extruded linseed	6%, 24–38	% total FA	129.5 *	6.3	-16.7	−20.0	−10.0	0.0	25.0	−55.9 *	[61]
Extruded linseed	9%, 24–38	% total FA	245.5 *	31.3	0.0	2.2	10.0	−10.2 *	−33.3	−58.8 *	[61]
Extruded linseed	5%, 15–26	% FAME	95.7 *	9.1	-60.0	−11.5	24.1 * ^4^	3.5	12.5	−52.0 *	[62]
Extruded linseed	10%, 15–26	% FAME	136.2 *	36.4	-60.0	−20.0 *	51.4 * ^4^	−2.9	12.5	−60.2 *	[62]
Extruded linseed	10.5%, 17–26	% FAME	247.2 *	121.1 *	25.0	6.9	75.5 * ^4^	−12.4 *	−15.4	−51.2 *	[63]
Chia seed	10%, 17–26	% FAME	226.4 *	89.5 *	25.0	6.4	47.0 * ^4^	−10.1 *	−3.8	−46.9 *	[63]
Linseed oil	6%, 40–57	% FAME	248.0 *	71.4	-25.0	0.0	85.3 *	−11.4 *	19.5	−66.9 *	[64]
Treated linseed ^5^	17.9%, 40–57	% FAME	412.0 *	328.6 *	100.0 *	−16.7	35.8	−5.0 *	72.0 *	−78.2 *	[64]
Linseed oil	4.8%, 20–36	mg FAME/100 g muscle	178.4 *	88.2 *	54.5 *	−1.8	30.8	−26.5 *	−2.3	−15.0	[65]
Extruded linseed	3%, 17–27	g/100 g muscle	121.9 *	−70.0	−33.3	7.4	−2.2	2.7	22.6	−22.1 *	[66]
Linseed oil	2.5%, 33, −(49 d ^3^)	g/100 g tissue	0.0	0.0	100.0	−23.9	-	5.1	-	−23.1 *	[67]
Linseed oil	5%, 33, −(49 d ^3^)	g/100 g tissue	33.3	33.3	100.0	−11.9	-	−2.7	-	−30.8 *	[67]
Extruded linseed	12.5%, 15–26	% total FA	555.3 *	252.9 *	140.9	16.2	-	−5.2	-	-	[68]
Ext. linseed + algae	10.7% + 4%, 15–26	% total FA	371.1 *	205.9 *	181.8	4.6	-	0.1	-	-	[68]
Fish oil	3.3%, −26	% total FA	−23.7	958.8 *	1104.5 *	−28.9	-	−14.1	-	-	[68]
Fish oil	3% fish oil, 28–46	% total FA	82.2 *	304.2 *	456.3 *	27.0	-	6.4	-	−64.8 *	[69]
Algae	2%, 15–26	% total FA	−2.8	658.8 *	1240.0 *	−11.7	-	−7.9 *	7.4	−79.2 *	[70]
Extruded linseed	10%, 16–26	% FAME	360.0 *	289.5 *	60.0	−12.7	144.4 * ^4^	−13.8 *	-8.3	−64.1 *	[71]
Ext. linseed + algae	5% + 3.9%, 16–26	% FAME	122.5 *	431.6 *	1880.0 *	−23.2	175.3 * ^4^	−26.3 *	33.3	−57.6 *	[71]
Linseed	10.7%, 35, −(56 d ^3^)	mg/100 g muscle	73.5 *	2.8	−11.8	10.7	-	-	-	−21.7 *	[48]
Linseed + algae	10.7% + 1.8%, 35, −(56 d ^3^)	mg/100 g muscle	−6.4	23.9 *	734.2 *	−6.7	-	-	-	−38.2 *	[48]
Algae	1.8%, 35, −(56 d ^3^)	mg/100 g muscle	20.4 *	−2.3	552.6 *	−2.4	-	-	-	−27.4 *	[48]
Algae	3%, 18–31	mg/100 g muscle	16.9	550.0 *	1578.6 *	−19.0 *	211.3 *	−5.2	94.7 *	−66.9 *	[72]
Algae	1.95%, 35, –	mg/100 g muscle	−9.8	51.7 *	491.7 *	1.7	-	−21.9 *	-	−31.4 *	[73]
Fish oil	1%, 30, −(30 d ^3^)	μg/g muscle	−31.2	-	-	−1.8	−37.0	-	300.0	-	[74]

Abbreviations: FA = fatty acid; FAME = fatty acid methyl ester. ^1^ Inclusion level = % of dry matter. The live weight of the animals is indicated as the initial and final live weight in kg. ^2^ C18:3*n*-3 = α-linolenic acid (ALA); C20:5*n*-3 = eicosapentaenoic acid (EPA); C22:6*n*-3 = docosahexaenoic acid (DHA); C18:2*n*-6 = linoleic acid (LA); C18:1*t*11 = vaccenic acid (VA); C18:1*c*9= oleic acid; C18:2*c*9*t*11= CLA; *n*-6/*n*-3 = *n*-6 PUFA/*n*-3 PUFA. ^3^ Experimental period in days (d). ^4^ C18:1*t*11 and C18:1*t*11 were unresolved and thus grouped. ^5^ Formaldehyde-treated whole linseed. * Indicates *p* < 0.05. A value for statistical significance of *p* < 0.05 was taken to indicate all cases in the same manner.

**Table 2:** Results of different studies of the effect of dietary inclusion of *n*-3 long chain polyunsaturated fatty acid (PUFA)-rich sources on the fatty acid composition of subcutaneous fat in ruminants. Effects are shown as relative variation with respect to the control diet and expressed in percentage.

	Inclusion Level, Live Weight ^1^	Unit	C18:3*n*-3 ^2^	C20:5*n*-3	C22:6*n*-3	C18:2*n*-6	C18:1*t*11	C18:1*c*9	C18:2*c*9*t*11	*n*-6/*n*-3	Reference
**Cattle**											
Whole linseed	8%, 500–700	% total FA	127.8 *	0.0	100.0	31.7 *	1.8	10.6 *	86.0 *	-	[52]
Cracked linseed	8%, 500–700	% total FA	50.0 *	25.0	100.0	64.5 *	9.9	10.8 *	88.0 *	-	[52]
Whole linseed	10%, 240–458	% total FA	690.0 *	-	-	0.5	9.1 ^4^	5.0	100.0 *	−83.0 *	[54]
Linseed oil	4.5%, 419, −(90 d ^3^)	% total FA	123.1 *	325.0 *	-	−28.3 *	-	−4.9	111.3 *	−63.1 *	[55]
Linseed oil	4.5%, −344	% total FA	163.3 *	-	-	−28.1 *	−30.8 *	0.3	−15.7	−72.0 *	[56]
Ground linseed	8%, 171–619	% total FA	88.0 *	-	-	−1.8	-	4.9	0.0	−48.7 *	[75]
**Sheep**											
Crushed linseed	10%, −38	% total FA	138.5 *	-	-	6.0	-	−24.2 *	110.3 *	−28.2	[58]
Linseed oil	2%, 23–50	% total FA	225.6 *	100.0 *	33.3	−39.4 *	0.5	3.1	−10.3	−79.7 *	[76]
Extruded linseed	3%, 24–38	% total FA	125.0 *	-	-	8.7	11.1	2.2	−28.6	−48.2 *	[61]
Extruded linseed	6%, 24–38	% total FA	200.0 *	-	-	8.7	−9.0	9.1 *	−21.4	−62.5 *	[61]
Extruded linseed	9%, 24–38	% total FA	375.0 *	-	-	30.4	−19.4	−3.4	1.4	−66.1 *	[61]
Extruded linseed	5%, 15–26	% FAME	71.9 *	−50.0	-	−31.2 *	23.0 * ^4^	−8.8 *	10.0	−55.3 *	[62]
Extruded linseed	10%, 15–26	% FAME	84.4 *	0.0	-	−40.1 *	33.4 * ^4^	−11.6	30.0	−60.6 *	[62]
Extruded linseed	10.5%, 17–26	% FAME	208.9 *	100.0 *	0.0	−21.1 *	60.3 * ^4^	−12.8 *	−28.6	−50.2 *	[63]
Chia seed	10%, 17–26	% FAME	180.0 *	100.0 *	0.0	−21.1 *	41.7 ^4^	−13.4 *	−9.5	−38.2 *	[63]
Linseed oil	6%, 40–57	% FAME	604.5 *	200.0 *	0.0	43.6	129.2 *	−15.0	76.3 *	−72.6 *	[64]
Treated linseed ^5^	17.9%, 40–57	% FAME	850.0 *	600.0 *	100.0	−2.0	59.0 *	−9.0	159.3 *	−87.0 *	[64]
Extruded linseed	10%, 16–26	% FAME	418.9 *	42.9	100.0	−37.4 *	101.4 * ^4^	−21.8 *	−6.3	−78.6 *	[71]
Ext. linseed + algae	5% + 3.9%, 16–26	% FAME	127.0 *	200.0 *	2800.0 *	−51.9 *	73.1 * ^4^	−24.6 *	−31.3 *	−66.1 *	[71]
Algae	1%, 19–46	mg/100 g tissue	35.0	35.3 *	504.8 *	−3.5	77.7	26.2	101.2	−85.4 *	[77]
Algae	2%, 19–46	mg/100 g tissue	43.2	264.7 *	1671.4 *	15.7	126.1	34.3	126.3	−79.5 *	[77]
Algae	3%, 19–46	mg/100 g tissue	58.1	411.8 *	1614.3 *	13.7	74.4	52.3	70.8	−79.9 *	[77]

Abbreviations: FA = fatty acid; FAME = fatty acid methyl ester. ^1^ Inclusion level = % of dry matter. The live weight of the animals is indicated as the initial and final live weight in kg. ^2^ C18:3*n*-3 = α-linolenic acid (ALA); C20:5*n*-3 = eicosapentaenoic acid (EPA); C22:6*n*-3 = docosahexaenoic acid (DHA); C18:2*n*-6 = linoleic acid (LA); C18:1*t*11 = vaccenic acid (VA); C18:1*c*9= oleic acid; C18:2*c*9*t*11= CLA; *n*-6/*n*-3 = *n*-6 PUFA/*n*-3 PUFA. ^3^ Experimental period in days (d). ^4^ C18:1*t*11 and C18:1*t*11 were unresolved and thus grouped. ^5^ Formaldehyde-treated whole linseed. * Indicates *p* < 0.05. A value for statistical significance of *p* < 0.05 was taken to indicate all cases in the same manner.

**Table 3:** Results of different studies of the effect of dietary inclusion of *n*-3 long chain polyunsaturated fatty acid (PUFA) rich sources on the adipogenic and lipid metabolism gene expression in ruminants.

	Inclusion Level, Final Live Weight ^1^	Tissue	Major Findings ^2^											Reference
			*PPARG*	*PPARA*	*CEBPA*	*SREBP1*	*ACACA*	*FASN*	*LPL*	*SCD*	*FADS1*	*FADS2*	*ELOVL5*	
**Cattle**														
Ground linseed	907 g/d, 107 d ^4^	*LD*	↑	≈	-	-	-	-	-	-	-	-	-	[127]
Extruded linseed	4.45%, 105 d ^4^	*LD* ^5^	-	≈	-	≈	-	-	-	-	≈	≈	≈	[128]
Extruded linseed	4.45%, 105 d ^4^	*LD* ^5^	-	↑	-	↑	-	-	-	-	↑	↑	≈	[128]
Extruded linseed	4.45%, 105 d ^4^	*LD* ^5^	-	↑	-	≈	-	-	-	-	↑	↑	≈	[128]
Whole linseed	8%, 700	*LT*	↑	-	-	≈	↓	↓	↑	↓	-	-	-	[52]
Cracked linseed	8%, 700	*LT*	↑	-	-	↓	↓	↓	↑	↓	-	-	-	[52]
Ground linseed	8%, 619	SC	≈	-	-	-	-	≈	↓	↓	-	-	-	[75]
Grass silage ^3^	630	*LD*	-	-	-	≈	↓	↓	-	↓	≈	≈	-	[140]
Grass silage ^3^	630	SC	-	-	-	↓	↓	↓	-	≈	≈	≈	-	[140]
Grass silage ^3^	625	*LD*	-	-	-	≈	≈	≈	-	≈	-	-	-	[141]
Algae	0.85%, 125 d ^4^	*SC*	≈	-	-	≈	≈	≈	≈	≈	≈	≈	-	[137]
Fish oil	0.85%, 125 d ^4^	*SC*	≈	-	-	≈	≈	≈	≈	≈	≈	≈	-	[137]
Fish oil	1%, 100 d ^4^	*LT*	-	-	-	-	-	-	-	↓	-	-	-	[142]
Fish oil	2%, 100 d ^4^	*LT*	≈	-	-	↓	-	-	-	↓	-	-	-	[142]
Fish oil	1%, 720	SC	≈	-	-	≈	≈	≈	↑	↑	≈	-	-	[135]
**Sheep**														
Extruded linseed	5%, 26	*LD*	-	-	-	-	↓	-	↑	≈	↓	↓	-	[62]
Extruded linseed	10%, 26	*LD*	-	-	-	-	↓	-	≈	↓	↓	≈	-	[62]
Extruded linseed	5%, 26	SC	-	-	-	-	≈	-	↑	≈	≈	≈	≈	[62]
Extruded linseed	10%, 26	SC	-	-	-	-	↓	-	≈	↓	≈	≈	≈	[62]
Extruded linseed	10.5%, 26	*LD*	-	-	-	-	↓	-	≈	↓	≈	↓	-	[63]
Chia seed	10%, 26	*LD*	-	-	-	-	↓	-	↓	↓	↓	↓	-	[63]
Extruded linseed	10.5%, 26	SC	-	-	-	-	≈	-	≈	≈	↓	↓	↓	[63]
Chia seed	10%, 26	SC	-	-	-	-	≈	-	≈	≈	≈	↓	↓	[63]
Linseed oil	0.1% of BW	*LD*	-	-	-	≈	≈	↓	-	↓	-	-	-	[25]
Linseed oil	6%, 56 d ^4^	*LD*	-	-	-	-	-	-	-	↓	-	-	-	[143]
Extruded linseed	10%, 26	*LD*	≈	-	≈	≈	↓	-	≈	↓	↓	↓	-	[71]
Ext. linseed + algae	5% + 3.9%, 26	*LD*	≈	-	≈	≈	↓	-	↓	↓	↓	↓	-	[71]
Extruded linseed	10%, 26	SC	↑	-	↑	≈	↓	-	≈	↓	≈	≈	≈	[71]
Ext. linseed + algae	5% + 3.9%, 26	SC	↑	-	↑	≈	↓	-	↓	↓	≈	≈	≈	[71]
Algae	3%, 31	*LT*	↑	↑	-	↓	-	≈	-	↓	≈	↓	≈	[72]
Algae	1.92%	*LL*	-	-	-	-	≈	-	-	≈	≈	≈	-	[144]
Algae	1.92%	SC	-	-	-	-	≈	-	-	≈	≈	≈	-	[144]
EPA+ DHA ^6^	0.39%, 53	SC	≈	≈	-	-	-	≈	≈	≈	≈	≈	≈	[136]
**Goat**														
Linseed oil	1.3%, 100 d ^4^	SC	↑	↑	-	-	-	-	-	↓	-	-	-	[139]
Linseed oil	1.3%, 100 d ^4^	*ST*	↑	≈	-	-	-	-	-	↓	-	-	-	[138]
Linseed oil	1.3%, 100 d ^4^	*LD*	↑	↑	-	-	-	-	-	↓	-	-	-	[145]

Abbreviations: ACACA = acetyl-CoA carboxylase; BW = body weight; CEBPA = CAAT/enhancer-binding protein alpha; ELOVL5 = fatty acid elongase 5; FADS1 = fatty acid synthase 1; FADS2 = fatty acid synthase 2; FASN = fatty acid synthase; LL = Longissimus lumborum; LPL = lipoprotein lipase; LT = Longissimus thoracis; PPARA = peroxisome proliferator-activated receptor alpha; PPARG = peroxisome proliferator-activated receptor gamma; SC = subcutaneous; SCD = sterol-CoA desaturase; SREBP1 = sterol regulatory element-binding protein 1; ST = Semitendinosus. ^1^ Inclusion level = % of dry matter. The final live weight is indicated in kg. ^2^ Arrows indicate up (↑) or down-regulated (↓) genes compared to control. The almost equal sign (≈) symbolize no change in expression between control and n-3 diet. ^3^ The control group consisted of maize silage with soybean-based concentrate (n-6 PUFA) vs. a grass-silage-based intervention diet (n-3 PUFA). ^4^ Final live weight was not reported and experimental period in days (d) is indicated instead. ^5^ The studied animal breed were Limousin, Angus and Blonde d’Aquitaine, respectively. ^6^ Ca salt of EPA and DHA.

## Supplementary Materials

Supplementary materials can be found at https://www.mdpi.com/1422-0067/21/9/3183/s1. Table S1: Pearson’s correlation coefficients between fat cell size and number and main categories of fatty acids of the subcutaneous adipose tissue. Table S2: Effect of linseed (10%) and linseed (5%) + marine algae (3.89%) on G3DPH activity in the subcutaneous adipose tissue of lambs.

## Abbreviations

AA	Arachidonic acid
*ACACA*	*Acetyl-CoA carboxylase alpha*
ACC	Acetyl-CoA carboxylase
ACL	ATP-citrate lyase
ACOX1	Acyl-CoA oxidase
ACS	Acyl-CoA synthetase
ALA	α-linolenic acid
AP-1	Activator protein-1
AT	Adipose tissue
*CD36*	*Fatty acid translocase*
*CEBPA*	*CAAT/enhancer binding protein alpha*
*CEBPB*	*CAAT/enhancer binding protein beta*
*CEBPD*	*CAAT/enhancer binding protein delta*
ChREBP	Carbohydrate response element binding protein
CLA	Conjugated linoleic acid
*CPT1A*	*Carnitine Palmitoyltransferase 1A*
DHA	Docosahexaenoic acid
DHAP	Dihydroxyacetone phosphate
*DLK-1*	*Delta-like 1 homolog*
DMI	Dry matter intake
DPA	Docosapentaenoic acid
*ELOVL5*	*Fatty acid elongase 5*
EPA	Eicosapentaenoic acid
*FABP4*	*Fatty acid-binding protein 4*
*FADS1*	*Fatty acid desaturase 1*
*FADS2*	*Fatty acid desaturase 2*
FAME	Fatty acid methyl ester
FAO	Food and Agriculture Organization of the United Nations
FAS	Fatty acid synthase
*FASN*	*Fatty acid synthase*
FATP	Fatty acid transport protein
FATP1	Fatty acid transport protein
FFARs	Free fatty acid receptors
G3P	Glycerol-3-phosphate
G3PDH	Glycerol-3-phosphate dehydrogenase
G6PDH	Glucose-6-phosphate dehydrogenase
GATA	GATA binding proteins
GK	Glucokinase
GLUT4	Glucose transporter type-4
ICDH	Isocitrate dehydrogenase
IM	Intramuscular
LA	Linoleic acid
LCPUFA	Long chain polyunsaturated fatty acid
*L-PBE*	*Peroxisomal enoyl-CoA hydratase*
*LPL*	*Lipoprotein lipase*
ME	Malic enzyme
MUFA	Monounsaturated fatty acid
NADPH	Nicotinamide adenine dinucleotide phosphate
*PDK4*	*Pyruvate dehydrogenase kinase 4*
*PEPCK*	*Phosphoenolpyruvate carboxykinase*
*PFKFB3*	*6-phosphofurcto-2-kinase/fructose-2,6-bisphosphatase 3*
*PPARA*	*Peroxisome proliferator-activated receptor alpha*
*PPARG*	*Peroxisome proliferator-activated receptor gamma*
PPARs	Peroxisome proliferator-activated receptors
PUFA	Polyunsaturated fatty acids
*RXR*	*Retinoic X receptor*
*RXRA*	*Retinoic X receptor alpha*
SC	Subcutaneous
*SCD*	*Stearoyl-CoA desaturase*
SER	Sterol regulatory element
SFA	Saturated fatty acids
*SREBP1*	*Sterol regulatory element binding protein 1*
SREBPs	Sterol regulatory element binding proteins
TG	Triacylglyceride
VA	Vaccenic acid
VLDL	Very low density lipoproteins
WHO	World Health Organization
*WNT*	*Wingless-type MMTV integration site family members*
*ZFP423*	*Zinc finger protein 423*
6PGD	6-phosphogluconate dehydrogenase
