# Supplementary material for: Adipose Tissue Modification through Feeding Strategies and Their Implication on Adipogenesis and Adipose Tissue Metabolism in Ruminants
Source: Int J Mol Sci. 2020 Apr 30;21(9):3183. doi: 10.3390/ijms21093183 (PMC7246642; doi:10.3390/ijms21093183)
Supplement: Supplementary file 1 [file ijms-21-03183-s001.pdf]

Supplementary Materials

Journal:
International Journal of Molecular Sciences

Title:
Adipose tissue modification through feeding strategies and their implication on adipogenesis and adipose tissue metabolism in ruminants

Authors:
Olaia Urrutia*, José Antonio Mendizabal, Leopoldo Alfonso, Beatriz Soret, Kizkitza Insausti and Ana Arana

Affiliation:
[1] IS-FOOD Institute, Escuela Técnica Superior de Ingeniería Agronómica y Biociencias, Departamento de Agronomía, Biotecnología y Alimentación, Universidad Pública de Navarra, Pamplona, Spain

*** Corresponding author:**
olaia.urrutia@unavarra.es

Supplementary Material 1. Reanalyses of the raw data from Urrutia et al. [1]: Methodology.

Raw data of intramuscular and subcutaneous adipocyte size of lambs from Urrutia et al. [1] were reanalyzed using AdipSD software, freely available in www.unavarra.es/rmga/add/. This software computes normalised histograms for the distribution of adipocyte size, tests the unimodality of the distribution, and computes different statistical descriptive parameters of the distributions depending on its uni or bimodality.

In the reanalyses, first, probability density functions were plotted for intramuscular and subcutaneous adipocyte size of the groups of lambs fed with control diet and linseed, and linseed and algae addition (Figure 5 of the main text).

Second, unimodality of adipocyte size distribution was tested for each tissue. For subcutaneous adipose tissue, unimodality was rejected and the existence of two adipocyte populations was assumed ('small' and 'large' adipocytes populations). Consequently, the first and second modes, the nadir (the low point in frequency between the two adipocyte populations), the % of adipocytes above nadir, the number of small and large adipocytes, their ratio, and the volume of small and large adipocytes were calculated. These parameters were computed for each animal, and then Pearson's correlation coefficients with main categories of fatty acids calculated (SFA, MUFA, *n*-3 PUFA, *n*-6 PUFA, *n*-6/*n*-3, PUFA/SFA, ALA, EPA, DHA) (results in Supplementary Material 2).

Supplementary Material 2. Reanalyses of the raw data from Urrutia et al. [1]: Results.

Table S1. Pearson´s correlation coefficients between fat cell size and number and main categories of fatty acids of the subcutaneous adipose tissue.

		**Nadir (μm)[1]**	**%Above nadir**	**No. small/ No. large**	**1srt mode (μm)**	**2nd mode (μm)**	**No. small**	**Vol. small (pL)**	**No. large**	**Vol. large (pL)**
SFA	*r*	**-0.51703**	-0.00503	0.02561	-0.20375	-0.03628	0.22267	**-0.58551**	0.40427	-0.37178
	P	**0.0137**	0.9823	0.9099	0.3631	0.8726	0.3192	**0.0042**	0.062	0.0884
MUFA	*r*	**0.44985**	0.03158	-0.05413	0.21554	0.15781	-0.23998	0.5038	**-0.42357**	0.40993
	P	**0.0357**	0.889	0.8109	0.3354	0.483	0.2821	0.0168	**0.0495**	0.0581
n-3 PUFA	*r*	0.37073	0.35985	-0.39928	0.09154	0.09961	**-0.44819**	**0.51187**	-0.23825	0.28645
	P	0.0894	0.1	0.0656	0.6854	0.6592	**0.0364**	**0.0149**	0.2856	0.1962
n-6 PUFA	*r*	0.34525	-0.31393	0.35553	-0.02242	-0.34382	0.20833	0.28844	-0.10795	0.04656
	P	0.1156	0.1548	0.1044	0.9211	0.1172	0.3522	0.193	0.6325	0.837
n-6/*n*-3	*r*	-0.24258	**-0.57355**	**0.62667**	-0.27748	-0.27832	**0.63106**	-0.37354	0.26319	-0.3341
	P	0.2767	**0.0053**	**0.0018**	0.2112	0.2098	**0.0016**	0.0868	0.2366	0.1286
PUFA/SFA	*r*	**0.48017**	0.02005	-0.02223	0.07443	-0.12456	-0.15788	**0.53862**	-0.2554	0.25176
	P	**0.0237**	0.9294	0.9218	0.742	0.5808	0.4828	**0.0097**	0.2513	0.2584
ALA	*r*	0.3999	0.31891	-0.35201	0.09704	0.07891	-0.41465	**0.53726**	-0.24829	0.2957
	P	0.0652	0.148	0.1081	0.6675	0.727	0.055	**0.0099**	0.2652	0.1815
EPA	*r*	-0.26358	0.23318	-0.2889	-0.04398	0.09466	-0.19654	-0.2369	0.0957	-0.12258
	P	0.2359	0.2963	0.1922	0.8459	0.6752	0.3807	0.2885	0.6718	0.5868
DHA	*r*	-0.16664	0.27608	-0.30009	0.02479	0.1992	-0.1765	-0.18717	0.04901	-0.00558
	P	0.4586	0.2136	0.1748	0.9128	0.3741	0.432	0.4042	0.8285	0.9803

Abbreviations: ALA = α-linolenic acid; EPA = eicosapentaenoic acid; DHA = docosahexaenoic acid; MUFA = monounsaturated fatty acid; PUFA = polyunsaturated fatty acid; SFA = saturated fatty acid.

[1]Bimodal distributions can be described by the following parameters: midway point between two cell populations (called nadir), first and second mode and the number of small adipocytes (below nadir) and large adipocytes (above nadir) [2].

Supplementary Material 3. Glycerol-3-phosphate dehydrogenase activity in the subcutaneous adipose tissue of lambs analysed by Urrutia et al. [1]: Unpublished results.

Table S2. Effect of linseed (10%) and linseed (5%) + marine algae (3.89%) on G3DPH activity in the subcutaneous adipose tissue of lambs.

	**Control**	**Linseed**	**Linseed + Algae**	**SEM**	***P*-value**
G3PDH, nmol·min-1·10^{-6} adipocytes					
Intramuscular	848	914	1112	202	0.668
Subcutaneous	155^{b}	263^{a}	282^{a}	33	0.026

^{a,b}Means with different lowercase superscripts within a row are different ($P < 0.05$).

Reference:

1. Urrutia, O.; Mendizabal, J.A.; Insausti, K.; Soret, B.; Purroy, A.; Arana, A. Effects of addition of linseed and marine algae to the diet on adipose tissue development, fatty acid profile, lipogenic gene expression, and meat quality in lambs. *PLoS ONE* **2016**, *11*, e0156765, doi:10.1371/journal.pone.0156765.
2. McLaughlin, T.; Sherman, A.; Tsao, P.; Gonzalez, O.; Yee, G.; Lamendola, C.; Reaven, G.M.; Cushman, S.W. Enhanced proportion of small adipose cells in insulin-resistant vs insulin-sensitive obese individuals implicates impaired adipogenesis. *Diabetologia* **2007**, *50*, 1707–1715, doi:10.1007/s00125-007-0708-y.
